# A Dual-Medium Model-Based Method for Rapid Thermal Performance Analysis of TPMS Porous Heat Exchange Structures

**DOI:** 10.3390/ma19204298

**Published:** 2026-10-12

**Authors:** Yuxin Hu, Yu Tian, Shutian Liu

**Affiliations:** State Key Laboratory of Structural Analysis for Industrial Equipment, Department of Engineering Mechanics, Dalian University of Technology, Dalian 116024, China; yuxinhu@mail.dlut.edu.cn (Y.H.); tianyv@mail.dlut.edu.cn (Y.T.)

**Keywords:** TPMS porous heat exchange structure, active heat transfer, dual-medium model, equivalent thermal performance, homogenization method

## Abstract

**Highlights:**

- A dual-medium model is proposed for constructing a rapid heat performance analysis method for TPMS structures.
- A TPMS structure is equivalent to a continuous solid thermal conductive medium and a coexistent Darcy–Forchheimer fluid medium.
- The thermal conductivity tensors of the equivalent solid and fluid are accurately calculated via the homogenization method.
- The volumetric heat transfer coefficient of a unit cell is calculated through numerical experiments using a one-dimensional flow model.
- This method improves computational efficiency by approximately 22 times while retaining high accuracy.

**Abstract:**

The triply periodic minimal surface (TPMS) structure demonstrates significant potential in the field of active heat transfer. This is due to its high specific surface area and controllable pore connectivity. However, its intricate geometric features result in notable computational costs during RANS-based CFD simulation, greatly limiting the efficiency of its design and optimization processes. Although existing equivalent approaches (e.g., the volume averaging method and the porous media model) have reduced the computational burden to some extent, they still suffer from limitations. Theseinclude insufficient sensitivity to microstructural features and low accuracy in characterizing convective heat transfer. In order to address the temperature-field prediction biases caused by the above limitations when filling TPMS on a large scale, this paper proposes a rapid analytical method based on a dual-medium model. The core idea of this method is to idealize the TPMS structure as a continuous solid thermal conductive medium and to homogenize its internal flow channels into a coexistent Darcy–Forchheimer fluid medium. The thermal conductivity tensor of the equivalent solid is accurately calculated via the homogenization method, whereas convective heat transfer in the fluid medium is characterized using empirical correlation equations. Ultimately, a rapid analytical model coupling solid heat conduction and fluid convection is developed at the macroscopic scale. Numerical examples show that, compared with traditional computational fluid dynamics (CFD) methods, this method improves computational efficiency by approximately 22 times while retaining high accuracy, thus providing an effective tool for the rapid design and performance assessment of TPMS-based active heat transfer structures.

## 1. Introduction

As thermal loads in engineering systems continue to increase, there is a growing need for compact cooling structures capable of removing heat under high heat flux conditions. Therefore, it is imperative to develop a high heat flux density heat transfer structure for it. Meanwhile, the TPMS exhibits zero mean curvature at every point, offering excellent smoothness, high specific surface area, adjustable porosity and superior heat transfer synergy. These key characteristics enhance fluid flow stability and heat transfer efficiency, positioning it as a promising candidate for active heat transfer applications. It is worth noting that the heat dissipation performance of the TPMS structure strongly depends on its geometric configuration, including surface topology, relative density, and cell size. Therefore, optimizing the design of TPMS-based cooling systems requires a fast and accurate method for predicting and analyzing heat transfer performance.

Extensive literature confirms that TPMS structures excel in the field of active heat transfer, establishing them as a global research focus [1,2,3,4]. Chinese research teams have conducted in-depth investigations in this area. For instance, Zhang et al. [5,6,7] developed four porosity-graded TPMS models (with porosity levels of 17.5%, 15%, 10%, and 5%) covering Gyroid, I-WP, and Primitive topologies, and numerically studied the thermal performance of phase change material (PCM)-embedded gradient TPMS structures. Their findings revealed that the thermal conductivity in the initial stage increases with the magnitude of the porosity gradient, but declines in the later stage compared to uniform structures. Wang et al. [8,9,10] developed a hybrid active–passive cooling system for electronic devices, combining TPMS structures with PCMs, and observed stable periodic variations in base temperature, liquid fraction, and Grashof number during intermittent operation. The above two scholars have studied and developed the important value of TPMS in its interaction with PCM. Wang et al. [11,12] proposed a dual-header heat sink design to improve flow uniformity in TPMS-based heat exchangers, demonstrating that reducing channel porosity enhances flow distribution. Tang et al. [13,14] found that the Diamond, Gyroid, and I-WP TPMS models achieved better convective heat transfer performance than the Fins model at the cost of higher flow resistance.

Internationally, researchers have also explored the active heat transfer performance of TPMS structures. Gado’s research group [15,16,17] focused on thermal management of electronic components using PCM-TPMS hybrid heat sinks, comparing the heat transfer performance of Gyroid, I-WP, and Primitive-based configurations with varying relative densities (10%, 20%, and 30%). They concluded that rigid PCM-TPMS radiators yield the most significant base temperature reduction, with a slight performance improvement as relative density increases from 10% to 30%. Qureshi et al. [18,19,20] utilized TPMS-based metal lattices as thermal conductivity enhancers for organic PCMs, demonstrating that TPMS-PCM composites outperform pure PCMs. In a subsequent study [21], they compared the heat transfer performance of TPMS-based finned metal foams (Gyroid, I-WP, Primitive) with traditional metal foams, finding that TPMS structures reduce PCM melting time and increase average heat transfer coefficients via enhanced conduction and natural convection. The above-mentioned foreign scholars have made significant contributions in applying TPMS to accelerate PCM melting. Kruzel et al. [22,23,24,25] developed an indirect method for determining heat transfer coefficients in Gyroid-based compact heat exchangers, enabling measurements in boiling/condensing two-phase flows without disrupting the internal flow field. Iwaniszyn et al. [26,27,28] evaluated the effect of cell diameter on pressure drop and heat transfer in SLM-fabricated solid Gyroid structures, and proposed a new correlation for flow resistance and heat transfer. Park et al. [29] introduced a novel topology optimization framework that leverages homogenized triply periodic minimal surfaces (TPMS) as tunable building blocks, and the optimized geometries achieve superior performance across the Pareto frontier. Ohtani et al. [30] proposed a model that enables macroscopic evaluation of wall thickness effects on HX performance without resolving the complex TPMS geometry, while reducing the computational cost by approximately 260 times compared with full-scale simulations. The above four scholars have made significant contributions in the field of heat transfer in TPMS heat exchangers.

Despite these advances, existing research methods are still limited in the large-scale integration of TPMS structures to quickly and accurately predict the resulting temperature field distribution. Current research mostly uses RANS-based CFD simulation to fully reveal local flow and heat transfer mechanisms, but it is not friendly to the design of large-scale TPMS lattice porous structures. Previous work has mainly demonstrated the usefulness of TPMS, but it still falls short in predicting large-scale temperature fields quickly. When we establish large-scale Gyroid-type TPMSs, the computational resources required are extremely large, and the calculation of surface temperature-field distribution is time-consuming. Therefore, we need to find an accurate and efficient equivalent method. Therefore, our research question is how to quickly calculate the surface temperature field of a structure consisting of a large number of TPMS unit cells when the fluid flows slowly.

For rapid temperature-field prediction, scholars have explored equivalent analysis methods for periodic porous structures. The volume averaging method [31,32,33] derives macroscopic continuity, momentum, and energy equations by averaging the governing equations over representative volume elements (RVEs), capturing complex structural effects through equivalent parameters such as permeability, inertial drag coefficient, and effective thermal conductivity. But the assumption of scale separation is difficult to strictly satisfy, and the size of the unit cell is often on the same order of magnitude as the cooling channel and component wall thickness, without scale separation. The porous medium model [34,35,36] treats lattice structures as homogeneous porous media, describing flow via Darcy’s law [37] or the Forchheimer equation [38] and incorporating empirical heat transfer correlations. However, the assumption of local thermal equilibrium is an important source of calculation error. The empirical heat transfer correlations are suitable for different flow rates but require an accurate equivalent model. Despite offering some computational simplification, these traditional methods suffer from three critical limitations: (1) insufficient equivalent accuracy (equivalent parameters, especially thermal conductivity, are insensitive to TPMS’s unique micro-topological features, causing substantial prediction biases across different configurations); (2) crude convective heat transfer characterization (overly simplified empirical correlations fail to capture the enhanced heat transfer induced by the high specific surface area of TPMS and its unique channel morphology, even though they provide some computational efficiency); and (3) improper multi-physics coupling simplification (the local thermal equilibrium (LTE) assumption commonly adopted in solid–fluid coupling introduces significant errors under high flow velocities or large disparities in solid/fluid thermal conductivity).

In summary, existing methods struggle to balance computational efficiency and analytical accuracy when predicting temperature fields on heat exchange structures that incorporate minimal surfaces on a large scale. Consequently, they cannot meet the fast and precise design requirements of heat transfer structures based on TPMS. To address these research gaps, this study proposes a rapid analysis method for TPMS active heat transfer structures based on a dual-medium model. This method idealizes the solid TPMS skeleton and internal fluid channels as two continuous media, employs rigorous homogenization theory, and uses a MATLAB R2024a-based solver to calculate equivalent performance parameters, thereby constructing a rapid analysis model at the macroscale. Section 2 provides a detailed introduction to the proposed rapid analysis method. Section 3 presents numerical examples and verification. Section 4 draws conclusions, and the final sections include funding support, acknowledgments, and references.

## 2. Rapid Analysis Method Description

### 2.1. Geometric Model Construction

The geometric configuration of TPMS can be described by the iso-surface equation:

(1) $$\begin{array}{l} \phi (x,y,z)=C,\;\\ x=2\pi X/L_{x},\;y=2\pi Y/L_{y},\;z=2\pi Z/L_{z}\; \end{array}$$

where ϕ(x,y,z) is a level set function that defines the type of the TPMS. X, Y and Z are Cartesian coordinates, and $L_{x},L_{y},L_{z}$ denote the unit cell length along the X, Y, and Z axes. *C* is the iso-surface shape parameter. Some typical types of TPMS, such as Gyroid, Schwarz P, and Diamond TPMS, are described as

(2) Gyroid:      sin (x)cos (y)+sin (y)cos (z)+sin (z)cos (x)=C

(3) Schwarz P:       cos (x)+cos (y)+cos (z)=C

(4) $$\mathrm{Diamond:}\;\;\;\;\;\;\;\begin{array}{l} \sin\;(x)\sin\;(y)\sin\;(z)+\sin\;(x)\cos\;(y)\cos\;(z)+\\ \cos\;(x)\sin\;(y)\cos\;(z)+\cos\;(x)\cos\;(y)\sin\;(z)=C \end{array}$$

The TPMS structure can be defined as

(5) $${\left(X,Y,Z\right)}^{Τ}=\left\{{\left(X,Y,Z\right)}^{Τ},\left|C-\frac{t}{2}\leq \phi (2\pi X/L_{x},y=2\pi Y/L_{y},2\pi Z/L_{z})\leq C+\frac{t}{2}\right.\right\}$$

where *t* represents the wall thickness of the unit cell.

### 2.2. Dual-Medium Model for Active Heat Transfer Analysis of TPMS

**Figure 1:**
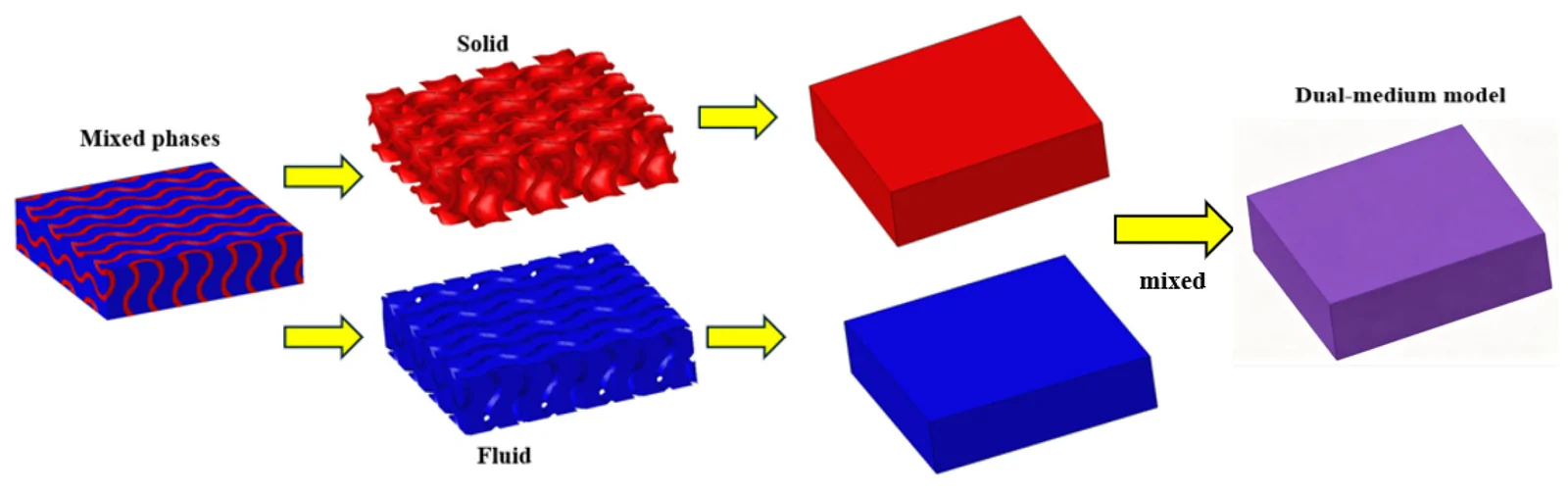
Schematic diagram illustrating the core idea of the rapid analysis method in this study.

The structure under analysis is a TPMS heat exchange configuration. For large-scale filling scenarios, the excessive computational cost makes it impractical to perform finite volume method simulations directly. To address this issue, a dual-medium equivalence strategy is proposed, as illustrated in Figure 1.

The proposed rapid analysis method is based on a dual-medium model. The core methodology involves partitioning the model into a solid domain and a fluid domain. Then, two continuous media are mixed to form a dual-medium model that coexists in the same space.

The solid domain ($\Omega_{s}$) is treated as a conductive medium continuously distributed in space, representing the TPMS solid skeleton. This assumption is proposed for the solid phase region of the equivalent dual-medium model of TPMS. In this domain, only heat conduction occurs, and the medium is characterized by an effective thermal conductivity tensor.

The fluid domain ($\Omega_{f}$) is treated as a Darcy–Forchheimer fluid medium continuously distributed in space, representing the internal gaps of the TPMS structure. Similarly, the above assumptions apply to the fluid phase part of the equivalent dual-medium model. This medium incorporates a heat source and accounts for both convection and heat conduction. Heat exchange between the solid and fluid domains occurs at their interface, thereby cooling the heated surface.

Based on the dual-medium model, we established a macroscopic governing equation system that couples flow and heat transfer. It is based on the assumption of a continuous medium, where the fluid satisfies conservation of mass, momentum, and energy.

Fluid domain equations

(6) $$\nabla \cdot (\rho_{f}u)=0\;\;\;\;u\in \Omega_{f}$$

(7) $$(\rho_{f}/\varepsilon )(u\cdot \nabla )u=-\nabla p+\nabla \cdot (\mu_{f}\nabla u)-(\mu_{f}\kappa^{-1}+\rho_{f}F\left.\left|u\right.\right|)u$$

(8) $$\rho_{f}c_{P,f}(u\cdot \nabla )T_{f}=\nabla \cdot (k_{f}^{eff}\nabla T_{f})+h_{v}(T_{s}-T_{f})\;\;\;T_{f}\in \Omega_{f}$$

(9) $$\frac{\partial (\rho k)}{\partial t}+\nabla \cdot (\rho \vec{u}k)=\nabla \cdot \left[\left(\mu_{f}+\frac{\mu_{t}}{\sigma_{k}}\right)\nabla k\right]+P_{k}-\beta^{*}\rho k\omega$$

(10) $$\frac{\partial (\rho \omega )}{\partial t}+\nabla \cdot (\rho \vec{u}\omega )=\nabla \cdot \left[\left(\mu_{f}+\frac{\mu_{t}}{\sigma_{\omega }}\right)\nabla \omega \right]+\alpha \frac{\rho }{\mu_{t}}P_{k}-\beta \rho \omega^{2}+D_{\omega }$$

(11) $$P_{k}=2\mu_{t}S_{ij}S_{ij},\;\;\;\;S_{ij}=\frac{1}{2}\left(\frac{\partial u_{i}}{\partial x_{j}}+\frac{\partial u_{j}}{\partial x_{i}}\right)$$

(12) $$D_{\omega }=2(1-F_{1})\frac{\rho }{\sigma_{\omega ,2}\omega }\nabla k\cdot \nabla \omega$$

Solid domain equation

(13) $$\nabla \cdot (k_{s}^{eff}\nabla T_{s})-h_{v}(T_{s}-T_{f})=0\;\;\;\;T_{s}\in \Omega_{s}$$

where Equations (6)–(8) respectively represent the continuity equation, the momentum conservation equation, and the energy conservation equation of the fluid domain. Equation (13) represents the energy conservation equation in the solid domain. Equations (9) and (10) represent the turbulence equations of the fluid domain, the SST k − ω model, here, k is turbulent kinetic energy and w is the specific dissipation rate and $\mu_{t}$ is the turbulent viscosity. $\sigma_{k}$ and $\sigma_{w}$ are the turbulent Prandtl numbers for the *k* and w equations, respectively. Subscripts 1 and 2 refer to the near-wall and outer-flow coefficients. The coefficients in the equations are taken as $\sigma_{k1}=1/0.85,\sigma_{k2}=1.0,\sigma_{\omega 1}=1/0.5=2.0,\sigma_{\omega 2}=1/0.856$. $P_{k}$ is the turbulent kinetic energy generation term; $\beta^{*},\alpha ,\beta$ are the closure coefficients of the SST, k−w model. They are taken as $\beta^{*}=0.09,\alpha_{1}=5/9,\alpha_{2}=0.44,\beta_{1}=0.075,\beta_{2}=0.0828$. $k_{f}^{eff},k_{s}^{eff}$ respectively represent the equivalent thermal conductivities of the fluid and solid phases of a TPMS cell under isotropy. $\rho_{f}$ is the fluid density, u is the macroscopic Darcy velocity, ε is the porosity, p is the pressure, $\mu_{f}$ is the fluid dynamic viscosity, κ is the permeability, F is the inertial drag coefficient, $c_{p,f}$ is the fluid specific heat capacity at constant pressure, $T_{f}$ is the fluid temperature, $T_{s}$ is the solid temperature, and $h_{v}$ is the volumetric heat transfer coefficient. The solid domain equation describes the thermal conductivity of solids and the heat transfer process between solids and fluids, and can be quickly solved for temperature fields on relatively rough grids using ANSYS Fluent 2024 R2.

### 2.3. Equivalent Performance of Equivalent Solid and Fluid Phase

The TPMS structure is treated as a composite material consisting of aluminum (solid phase) and water (fluid phase). The solid domain is equivalent to a continuous, isotropic thermally conductive medium, while the fluid domain is equivalent to a macroscopic continuous medium responsible for flow and convective heat transfer. Macroscopic equivalent parameters reflecting the microscopic characteristics of the TPMS are extracted via homogenization theory and numerical model experiments, providing data support for macro-scale analysis.

It should be noted that for a standard, undisturbed TPMS structure, due to its cubic symmetry, the thermal conductivity measured on the X, Y, and Z axes is exactly the same ($k_{xx}=k_{yy}=k_{zz}$); therefore, it can be considered isotropic in the classical sense of heat conduction. The calculated thermal conductivity values along the body diagonal direction usually have slight differences from those along the X-axis direction, and this difference can be completely ignored in the vast majority of engineering applications, so it is still classified as isotropic.

According to homogenization theory, the equivalent thermal conductivity coefficients of solid and fluid phases are more accurate when the number of cell fillings is large, but less accurate when the number of fillings is small. Moreover, for different microstructures such as pore size or topological structure, the method proposed in this paper still agrees well with the results of the fine finite volume method (FVM) analysis. See Section 3.3.5 for details.

#### 2.3.1. Effective Thermal Conductivity of Solid Phase

For the equivalent composite material of the TPMS unit cell [39], the following assumptions are adopted (consistent with homogenization theory for periodic porous structures [39]):

(a) The material is macroscopically uniform;
(b) The fluid and the solid substrate are continuously distributed with known thermal conductivities;
(c) The fluid is in full contact with the substrate, and contact thermal resistance is negligible (validated by Cheng and Liu [39]);
(d) The fluid exhibits a periodic distribution in the substrate (ensuring the unit cell can be extended to large-scale structures).

The characteristic equation solved on the TPMS unit cell is

(14) $$\nabla_{y}\cdot (k_{s,ij}(y)\nabla_{y}\Lambda_{j})=-\nabla_{y}\cdot (k_{s,ij}(y)e_{j})$$

where $k_{s,ij}$ is the intrinsic thermal conductivity tensor of the solid material, $\Lambda_{j}$ is the temperature disturbance function in the j-direction, and $e_{j}$ is the unit basis vector. Periodic boundary conditions are applied to the unit cell to solve for $\Lambda_{j}$, which is then substituted into the integral formula for effective thermal conductivity. For isotropic Gyroid structures, the formula simplifies to

(15) $$K_{s,ij}^{eff}=\frac{1}{\left|\left.Y\right|\right.}\int_{Y_{s}}k_{s,ij}(\delta_{ij}+\frac{\partial \Lambda_{j}}{\partial y_{i}})dY$$

where $K_{s,ij}^{eff}$ is the effective thermal conductivity tensor of the solid phase, Y is the domain occupied by the Gyroid unit cell, $Y_{s}$ is the domain occupied by the solid part, and $\delta_{ij}$ is the Kronecker delta. This approach is referred to the homogenization method [40].

#### 2.3.2. Effective Thermal Conductivity and Effective Fluid Performance of Fluid Phase

The solution logic for the fluid phase is consistent with that of the solid phase, with only the integration range and material thermal conductivity modified. The characteristic equation for solving $\Lambda_{j}$ is

(16) $$\nabla_{y}\cdot (k_{f,ij}(y)\nabla_{y}\Lambda_{j})=-\nabla_{y}\cdot (k_{f,ij}(y)e_{j})$$

The effective thermal conductivity tensor of the fluid phase is calculated as

(17) $$K_{f,ij}^{eff}=\frac{1}{\left|\left.Y\right|\right.}\int_{Y_{f}}k_{f,ij}(\delta_{ij}+\frac{\partial \Lambda_{j}}{\partial y_{i}})dY$$

where $k_{f,ij}$ is the intrinsic thermal conductivity tensor of the fluid, and Y_f_ is the domain occupied by the fluid part. Note that $\Lambda_{j}$ is defined over the entire unit cell domain Y, even though the integration is limited to Y_f_. A numerical solution for $\Lambda_{j}(y)$ is adopted due to the complexity of the Gyroid geometry.

Here, we treat the fluid in this article to a Darcy–Forchheimer fluid. The specific equivalent parameter calculation is provided in Section 3.2.

Additionally, the permeability *ĸ* and inertial drag coefficient *F* of the TPMS structure are obtained by solving the steady-state Forchheimer equations within a unit cell, which are used to describe fluid pressure loss in the macro-scale model.

(18) $$\Delta P=\frac{\mu }{\kappa }uL+F\rho_{f}u^{2}L$$

Formula (18) is the Darcy–Forchheimer formula, which represents the relationship between pressure drop and flow rate. After fitting the relationship between pressure drop and inlet flow rate, *ĸ* and *F* can be calculated based on the coefficients.

#### 2.3.3. Effective Volumetric Heat Transfer Coefficient Between Solid and Fluid Phase

**Figure 2:**
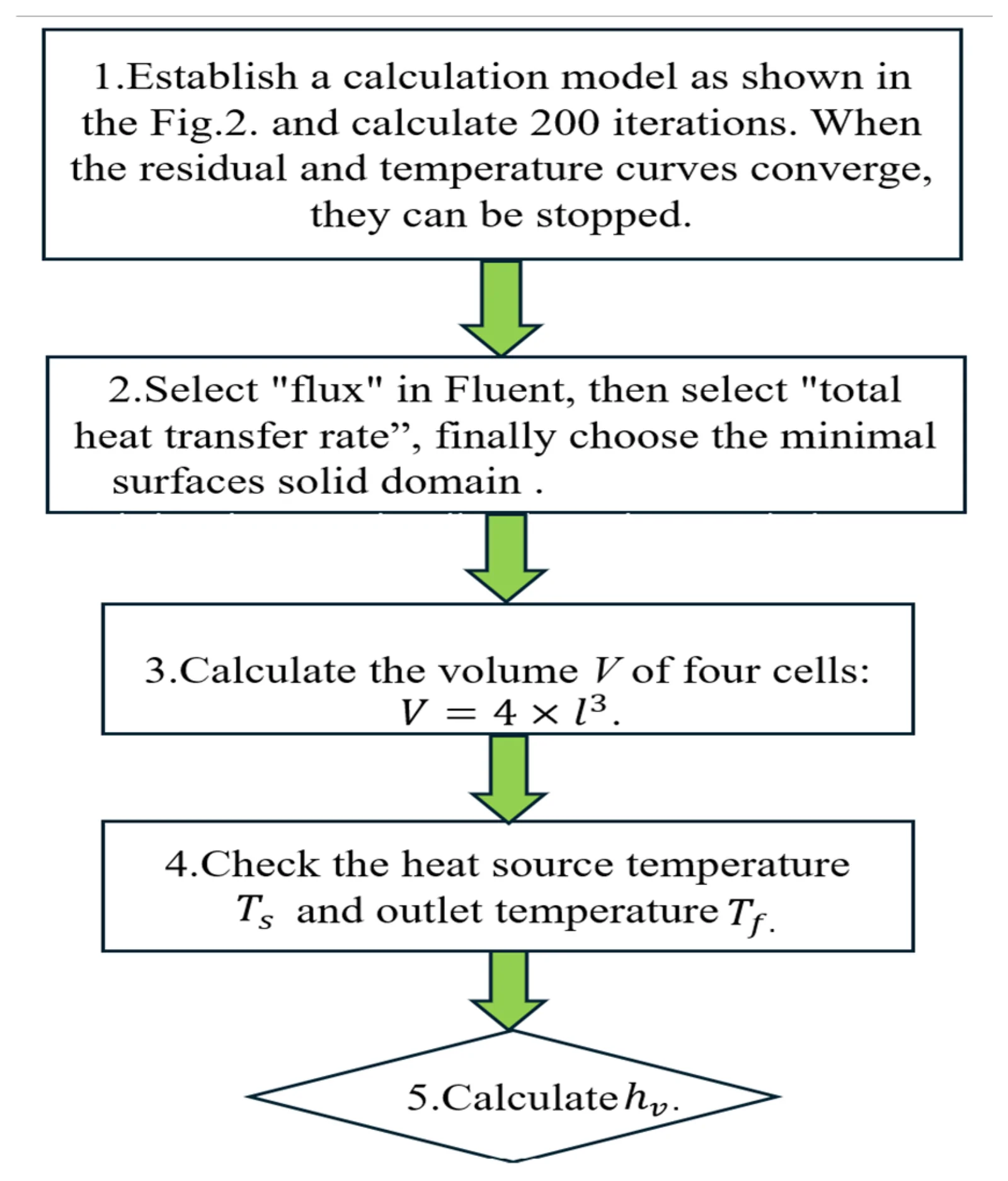
Calculation flowchart of the volumetric heat transfer coefficient $h_{v}$.

We have provided a general process for solving the volumetric heat transfer coefficient $h_{v}$ in Figure 2, which can be followed to solve $h_{v}$. For the volumetric heat transfer coefficient $h_{v}$, we define it using Equation (19), where $A_{q}$ is the heat transfer power per unit area, *V* is the volume of four cells, $T_{s}$ is the given unit cell temperature, and $T_{f}$ is the given fluid outlet temperature. The inlet fluid velocity is $v_{in}$.

(19) $$h_{v}=\frac{\iint A_{q}\cdot dS}{V\cdot (T_{s}-T_{f})}$$

**Figure 3:**
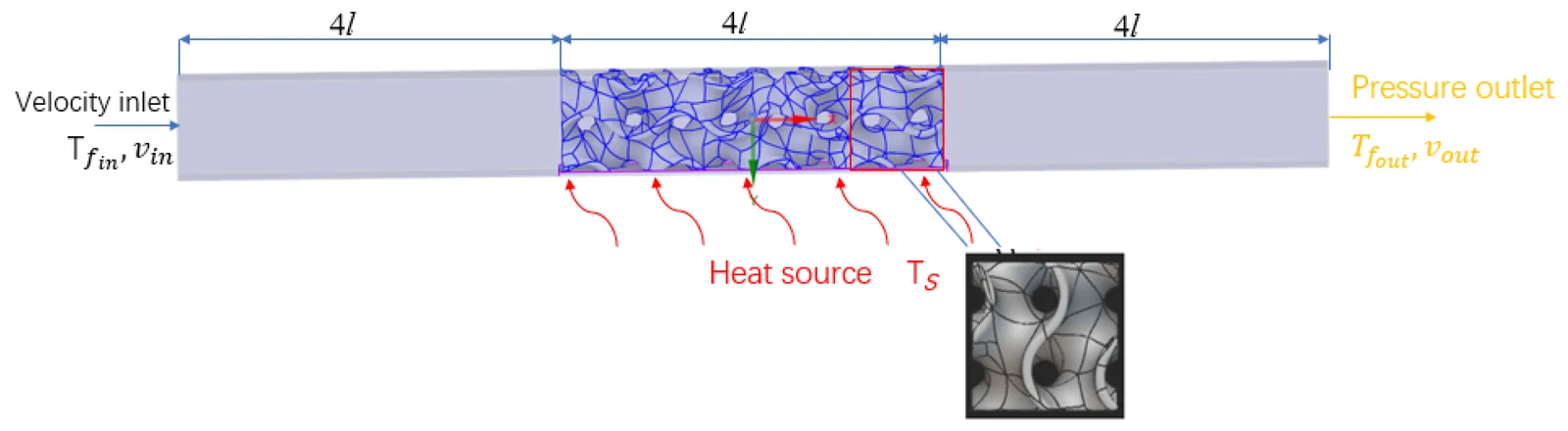
Schematic diagram of the numerical experiment for solving the model.

We solve it through numerical experiments of the model shown in Figure 3.

Model setup: Four Gyroid unit cells are placed at the center of a square tube, with 200 mm zones at both ends. The specific settings are shown in Figure 3. The geometry model parameters, material constants and boundary conditions refer to Table 1, Table 2 and Table 3. We give the four cells a temperature of $T_{s}$, a constant inlet fluid temperature, and an outlet temperature of $T_{f}$. We calculate the average volumetric heat transfer coefficient $h_{v}$ of the four cells, where *V* is the volume of the four cells. The $h_{v}$ expression is obtained from FVM model experiments. The $h_{v}$ expression represents the heat transfer power per unit volume and unit temperature rise.

Mesh generation: This compact heat Exchange [41] model is meshed in Fluent Meshing, with a grid independence analysis conducted to ensure accuracy (Section 3.1.2).

Calculation settings: The SST k-ω turbulence model was adopted, and the energy equation solver was enabled. The pressure-based Coupled algorithm was used for pressure–velocity coupling, with the momentum-based Rhie–Chow formulation selected for face mass fluxes. Gradients were evaluated using the least-squares cell-based method, and second-order pressure interpolation was employed. The convective terms in the momentum and energy equations were discretized using QUICK, whereas second-order upwind discretization was used for the turbulent kinetic energy and specific dissipation rate equations. Finally, we set appropriate convergence criteria.

### 2.4. Solution Process of the Equivalent Dual-Medium Model

(1) Determine the thermal conductivity properties of equivalent solids based on the type of triply periodic minimal surface structure.
(2) Determine the equivalent conductivity properties and equivalent fluid properties of the fluid.
(3) Determine the heat transfer coefficient between the fluid and solid phases.
(4) Use the finite volume method to solve the equivalent medium model (formula), and obtain $T_{s},T_{f},P,u$.

## 3. Numerical Examples and Verification

This section presents numerical validation of the temperature-field prediction accuracy and computational efficiency of the proposed dual-medium model-based rapid analysis method for Gyroid-type TPMS active cooling structures. First, key equivalent parameters of Gyroid-type TPMS unit cells are computed, and correlations describing their dependence on porosity and inlet flow velocity are established. The prediction accuracy is then systematically benchmarked against high-fidelity finite volume method (FVM) solutions under conditions across different filling scales, encompassing variable working conditions to demonstrate the universality of the method. Finally, the computational efficiency gains of the proposed method are quantified relative to the high-fidelity FVM approach.

**Figure 4:**
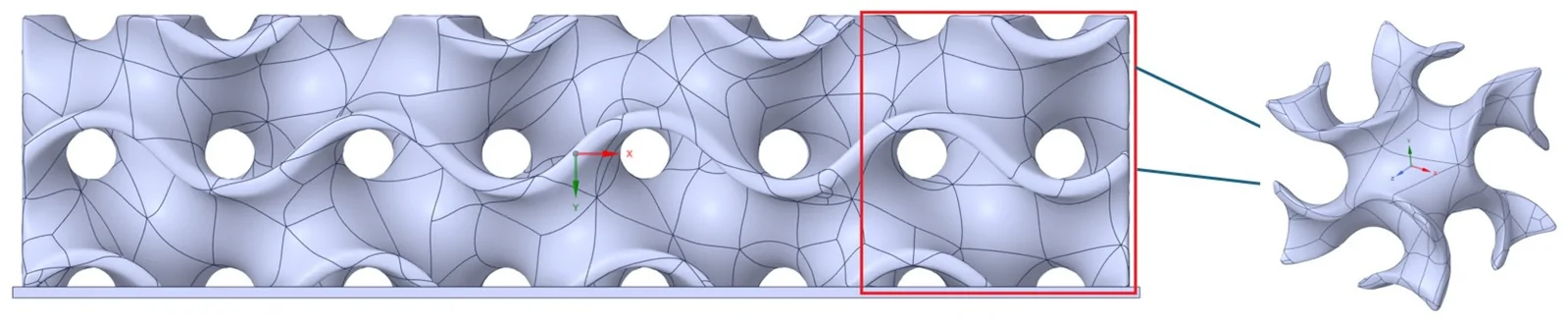
Schematic of a single Gyroid unit cell and a cluster of four cells.

Here, we take the Gyroid-type TPMS as an example. A set of Gyroid models was constructed using nTopology v4.12.2 software. The constructed basic unit cell and a cluster of four cells are shown in Figure 4.

We conduct a comparison of temperatures at the same measurement points and computational times between the results obtained by the finite volume method (FVM) in Fluent and the equivalent results of the same model calculated by the proposed method, to verify the accuracy and efficiency of the proposed method.

First, the effective parameters of the dual-medium model are calculated from the fundamental data of the unit cell. These parameters are then assigned to the rectangular computational domain to simulate water flow in the equivalent porous medium, and the computational accuracy and time cost of the proposed method are compared with those of the finite volume method (FVM). Finally, we simulate scenarios such as 4 × 4, 6 × 6, and 8 × 8 for filling calculations, proving that our method is still effective. You can refer to Section 3.3.1, Section 3.3.2 and Section 3.3.3 for the results.

### 3.1. Basic Unit Cell, Parameters and Mesh Independence Verification

In this section, we determine the geometric parameters of the base unit cell and the input material constants and boundary conditions for subsequent model construction, and perform the mesh independence verification of the FVM benchmark model to ensure the accuracy of the calculations.

#### 3.1.1. Basic Unit Cell Geometric Model

Comparison plan:

Fine FVM model: A full-scale model containing complete unit cells is constructed, and direct numerical simulation is performed to obtain the benchmark solution.

Rapid calculation model: Establish a macroscopic rectangular domain, assign its equivalent properties ($k^{\mathrm{eff}},\;\kappa ,\;F,\;h_{v}$) calculated by the method in Section 2, and perform macroscopic coupling solution.

The geometric model parameters, material constants, and boundary conditions are presented in Table 1, Table 2 and Table 3, respectively.

#### 3.1.2. Mesh Independence Verification

**Figure 5:**
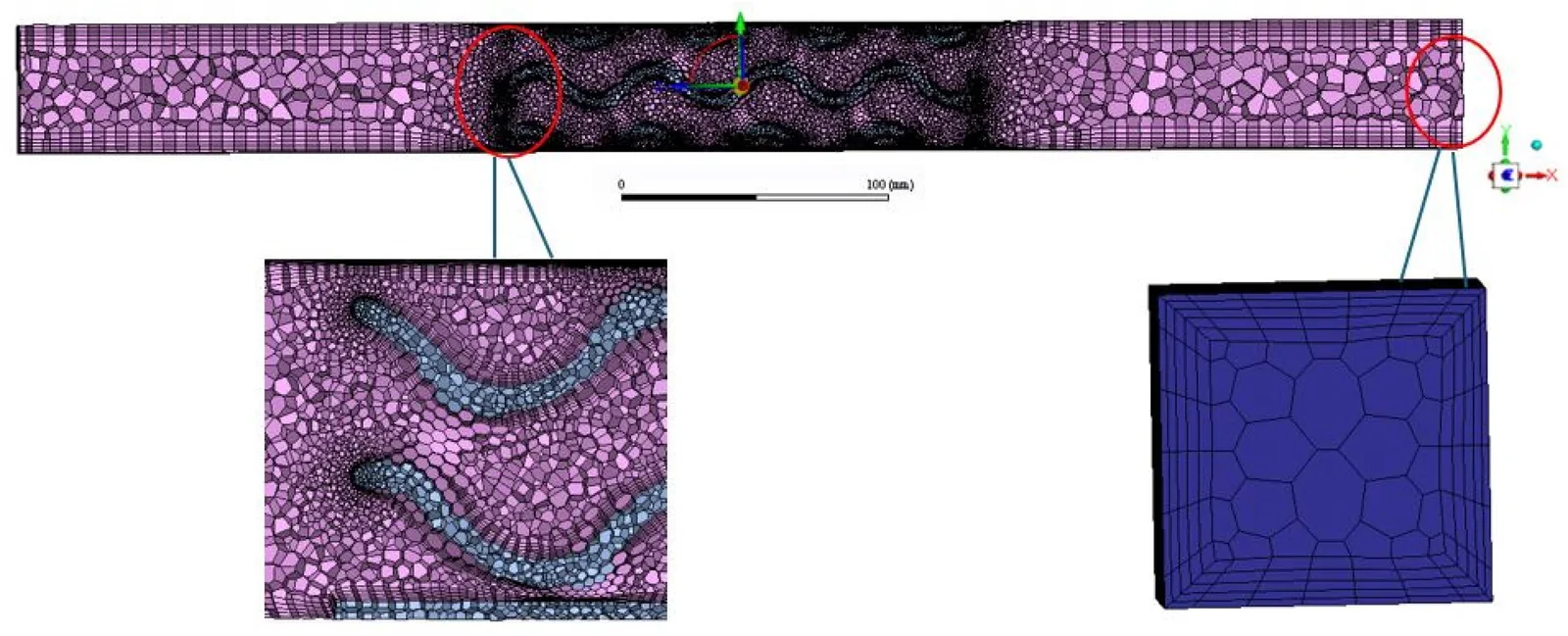
Mesh diagram of the standard Gyroid structure model.

The TPMS structure adopted herein is a network-type TPMS solid thickness structure modeled using nTopology. The default wall thickness is set to 5 mm with zero mid-surface offset, and the uniform period in all directions is 50 mm. The solid domain corresponding to the minimal surface consists of aluminum alloy, while the fluid domain (water) occupies the pore region of the TPMS geometry. The geometric model illustrated in Figure 3 is constructed and subsequently imported into Fluent Meshing for mesh sensitivity analysis. The resulting volume mesh is displayed in Figure 5. The convergence criteria for the continuity, momentum and energy equations are specified as 1 × 10^−6^.

**Figure 6:**
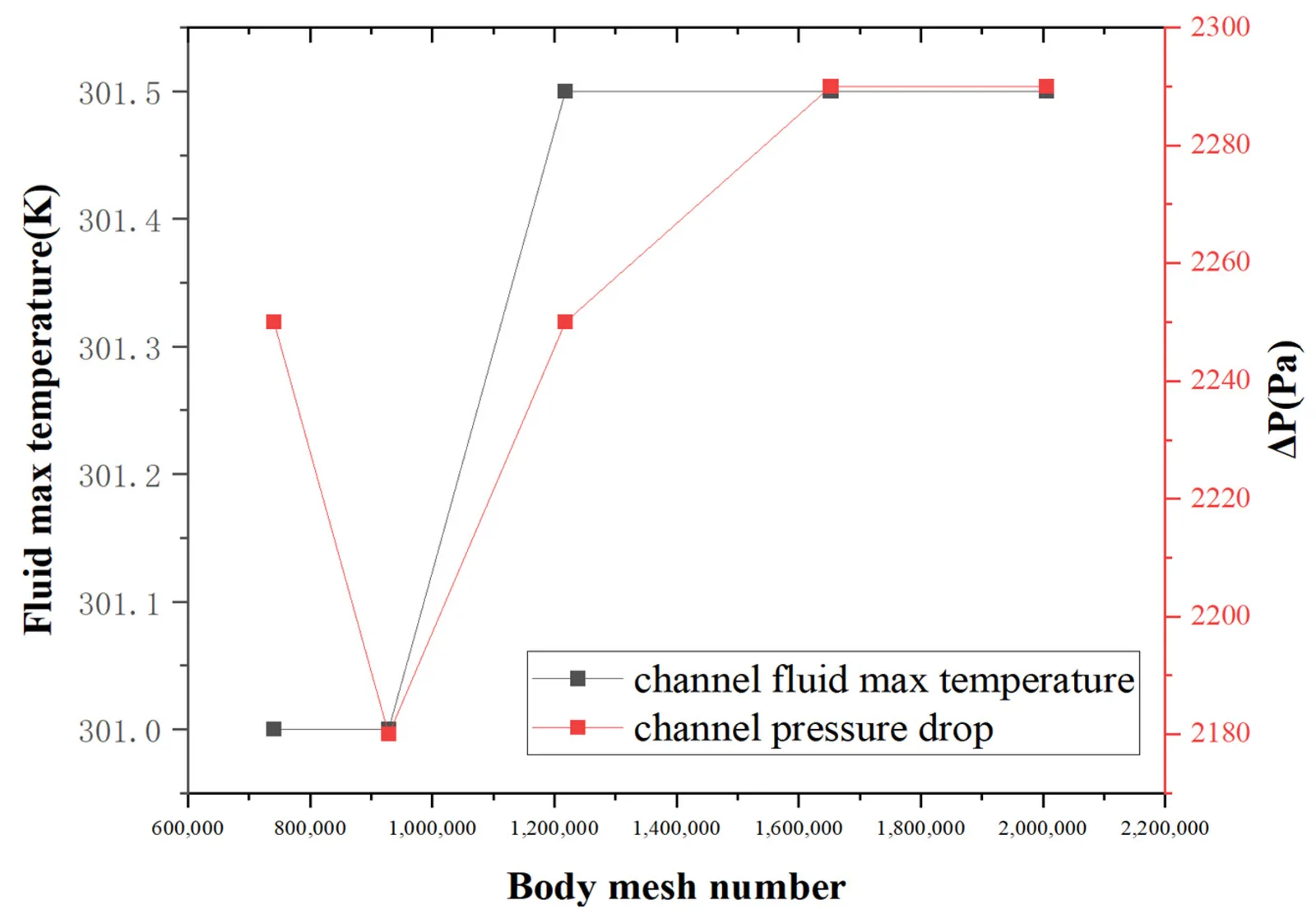
Convergence curves of outlet pressure and maximum temperature with respect to the number of mesh elements.

The number of mesh elements affects calculation accuracy and convergence time. Four cells mesh models are established with a maximum element size of 10 mm and minimum element sizes of 0.5 mm, 0.4 mm, 0.3 mm, 0.2 mm and 0.1 mm (corresponding to 740,597, 928,540, 1,217,750, 1,652,454 and 2,005,231 elements, respectively). The peak temperature ($T_{\max}$) and pressure drop (ΔP) of the Gyroid structure are calculated, with results shown in Figure 6.

As the number of mesh elements increases, the results gradually converge. When the number of elements reaches 1,652,454 (minimum element size = 0.2 mm), the changes in $T_{\max}$ and ΔP become negligible. A minimum element size of 0.2 mm and a maximum element size of 10 mm were adopted for these simulations. With this mesh setting, one Gyroid TPMS structure was discretized into 4,413,114 elements and achieved grid independence, as shown in Figure 6. Therefore, the same mesh-size settings were applied to subsequent structures formed by arraying the same unit cell.

### 3.2. Macro-Scale Coupling Analysis Model

#### 3.2.1. Model Establishment and Numerical Calculation in MATLAB

Based on the dual-medium model, a macro-scale governing equation system coupling flow and heat transfer is established. We developed a program using MATLAB R2024a software to solve the equivalent heat transfer coefficient tensor of the solid and fluid phases, as well as the volumetric heat transfer coefficient of single cell. Here we consider the standard Gyroid structure, which, due to its cubic symmetry, has identical thermal conductivity along the X, Y, and Z axes. The difference between the thermal conductivity calculated along the body diagonal and the thermal conductivity calculated along the x-axis is very small. Therefore, we consider it isotropic in the classical sense of thermal conduction. The results of the solution are as follows:

Solid phase: Equivalent density $\rho_{s}^{eff}$ = 689.85 kg/m^3^ (2700 kg/m^3^ × 25.55%), equivalent specific heat capacity $c_{p,s}^{eff}$ = 1800 J/(kg·K), effective thermal conductivity $K_{s}^{eff}$ = 42.4 W/(m·K) (calculated via the MATLAB program, and result shown in Table 4).

The equivalent solid here is a solid with a certain density and specific heat capacity at constant pressure. The equivalent parameter calculation of the equivalent solid is given by the following formulas:

(20) $$\rho_{s}^{eff}=2700\times 25.55\%=689.85\;{\mathrm{kg}/m}^{3}$$

(21) $$c_{p,s}^{eff}=c_{p,s}=1800\;J/(\mathrm{kg}\cdot K)$$

Fluid phase: The equivalent thermal conductivity coefficient $K_{f}^{eff}$ of the fluid is calculated using a MATLAB program. $K_{f}^{eff}=0.446426W/\left(m\cdot K\right)$, as shown in Table 5.

Volumetric heat transfer coefficient: Here, $\iint A_{q}\cdot dS$ is the total heat transfer rate from the Gyroid surface to the fluid (6720.5029 W for four cells), *V* = 125 × 4 × $10^{-6}$ $m^{3}$ (volume of four cells), $T_{s}$ = 373.15 K, and $T_{f}$ = 302 K. Substituting the values gives $h_{v}$ = 188,910.83 W/($m^{3}\cdot K$).

**Figure 7:**
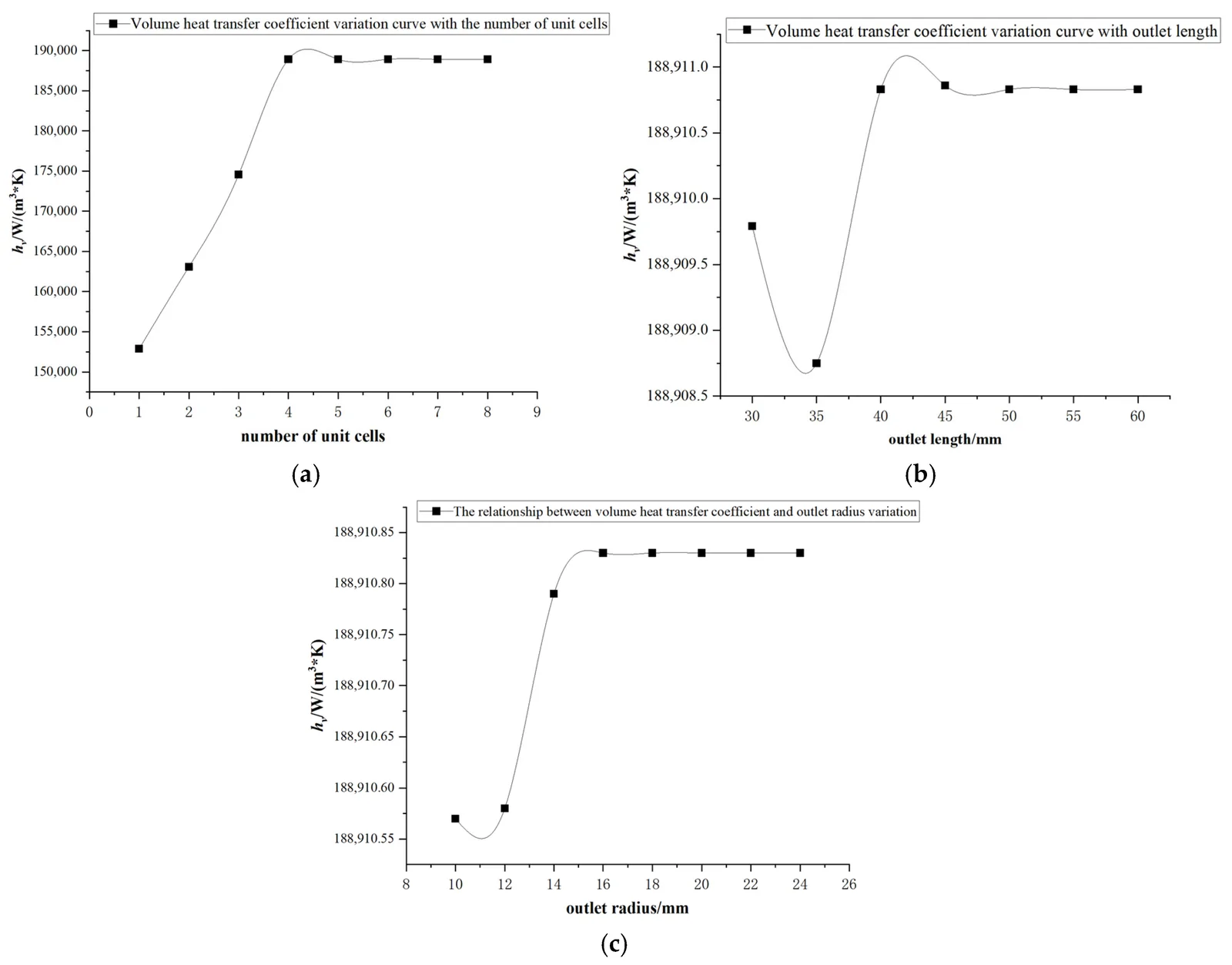
Sets of evidence of independence between $h_{v}$ and filling length and outlet boundary conditions. (**a**) $h_{v}$ vs. number of unit cells; (**b**) $h_{v}$ vs. outlet length; (**c**) $h_{v}$ vs. outlet radius.

The above $h_{v}$ is the volumetric heat transfer coefficient of each unit cell. As the number of one-dimensional filled cells increases, the average volumetric heat transfer coefficient of each unit cell first increases and then tends to converge. The convergence diagram is shown in Figure 7. Therefore, the volumetric heat transfer coefficient is independent of the length of the region; similarly, when we change the outlet diameter or outlet length, the volumetric heat transfer coefficient remains almost unchanged. Therefore, it is also unrelated to outlet conditions, as shown in the following figures.

**Figure 8:**
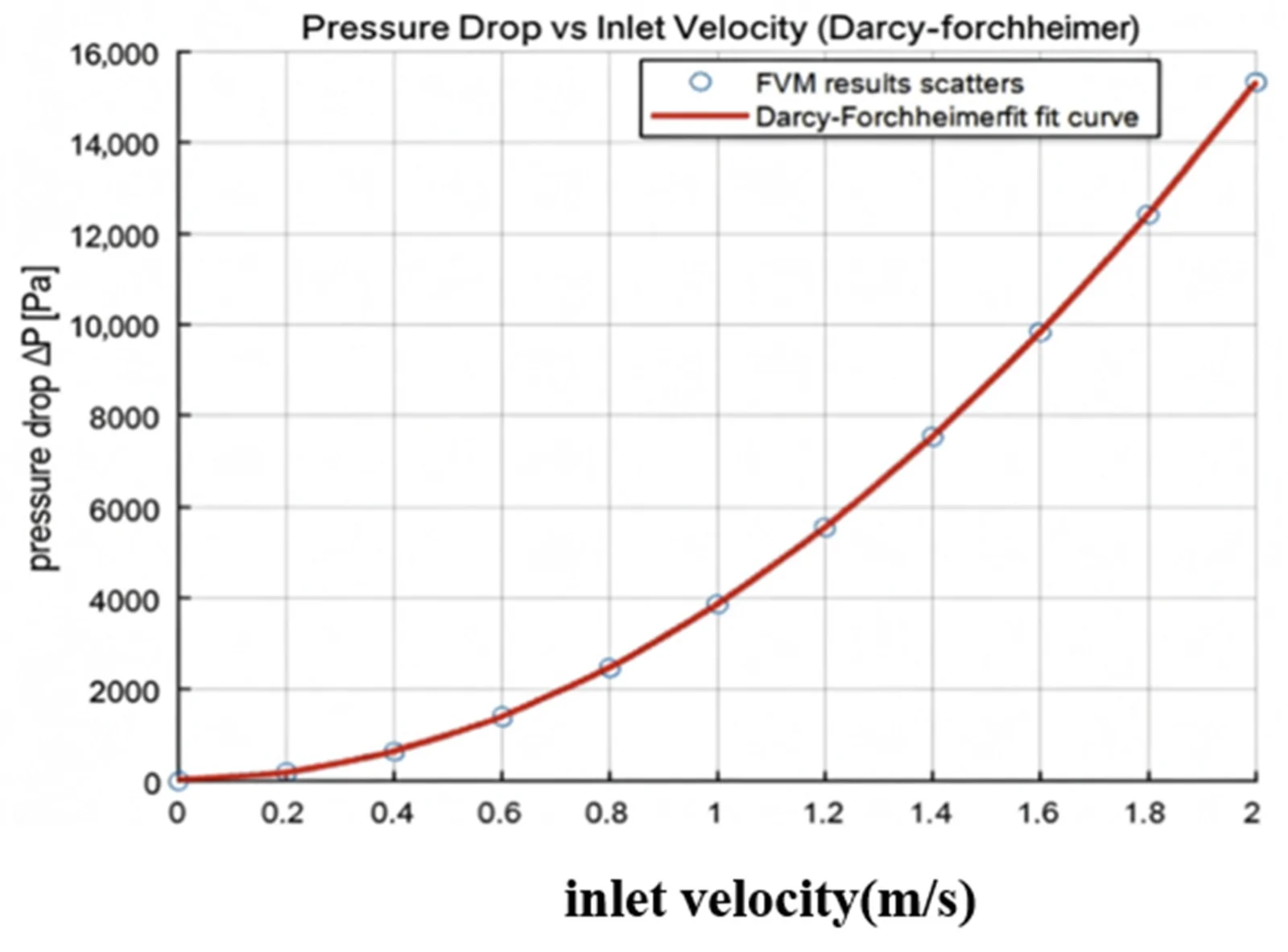
Filled 4 × 4 cell inlet pressure drop vs. flow velocity.

Flow parameters:

The permeability κ and inertial drag coefficient *F* are given by the relationship curve between pressure drop and flow velocity shown in Figure 8. The fluid pressure drop consists of two parts: viscous pressure drop and inertial pressure drop. The expression for pressure drop is given by the Darcy–Forchheimer law in Equation (18):

Permeability $\kappa =3.68\times 10^{-6}m^{2}$, inertial drag coefficient *F* = 19.07 $m^{-1}$ (fitted from the pressure drop vs. flow velocity curve, Figure 8).

The above parameters were obtained at a porosity of 74.45% and a flow rate of 0.4 m/s. When the porosity and flow rate change, we need to provide accurate expressions for the volumetric heat transfer coefficient permeability κ, and inertial drag coefficient *F*. We obtained them through Newton’s law of cooling. *K* and *F* are based on the classical general formula of the Ergun equation. Equation (29) refers to reference [42].

(22) $$Q=h_{v}\cdot V\cdot \Delta T=h\cdot a\cdot V\cdot \Delta T=h\cdot A\cdot \Delta T$$

(23) $$h_{v}=h\cdot a$$

(24) $$h=\frac{Nu\cdot K_{f}^{\mathrm{eff}}}{D_{h}}$$

(25) Nu=C⋅Rem⋅Prn

(26) $$h_{v}=71.33\cdot {\left(\frac{\rho_{f}u}{\mu }\right)}^{m}\cdot D_{h}^{m-1}\cdot K_{f}^{eff}\cdot a$$

(27) $$\kappa =\frac{\varepsilon^{3}d_{p}^{2}}{150{\left(1-\varepsilon \right)}^{2}}$$

(28) $$F=\frac{1.75(1-\varepsilon )}{\varepsilon^{3}d_{p}}$$

(29) $$\varepsilon =1-\frac{\pi }{6}[1-{\left(1-2\frac{t}{l}\right)}^{3}]$$

Here *h* is the convective heat transfer coefficient, a is the specific surface area of the surface, $D_{h}$ is the hydraulic diameter, *Nu* is the Nusselt number, $\mathrm{Pr}=\frac{\mu_{f}c_{p,f}}{k_{f}}$ is the Prandtl number of water, where $\mu_{f}$, $c_{p,f}$, and $k_{f}$ are the dynamic viscosity, specific heat capacity, and thermal conductivity of water, respectively. Its value is determined from the water properties used in the calculation rather than prescribed as a universal constant, and $d_{p}$ is the equivalent diameter of the unit cell.

#### 3.2.2. Calculation of Effective Parameters Under Different Porosities

We calculated the $h_{v}$ values at different flow rates and porosities using FVM in Fluent, and fitted these scatter points to obtain an accurate expression for $h_{v}$.

In the below four fitting graphs, each point is the result of FVM in Fluent and conforms to Formula (26). We chose polynomial fitting. From the graphs, it can be seen that $h_{v}$ almost always conforms to a quadratic function relationship, and the fitting curve has a 99.98% fit with the data points. Therefore, when the porosity is not 74.45%, we can use a quadratic relationship to accurately express the volumetric heat transfer coefficient $h_{v}$.

At the same time, as the porosity changes, the fluid pressure drop within the model also changes. We also conducted a statistical analysis of the pressure drop at different porosities and plotted a fitted data graph. These data points were all obtained through FVM in Fluent, where the permeability κ and inertial drag coefficient *F* both conform to Formulas (27) and (28).

**Figure 9:**
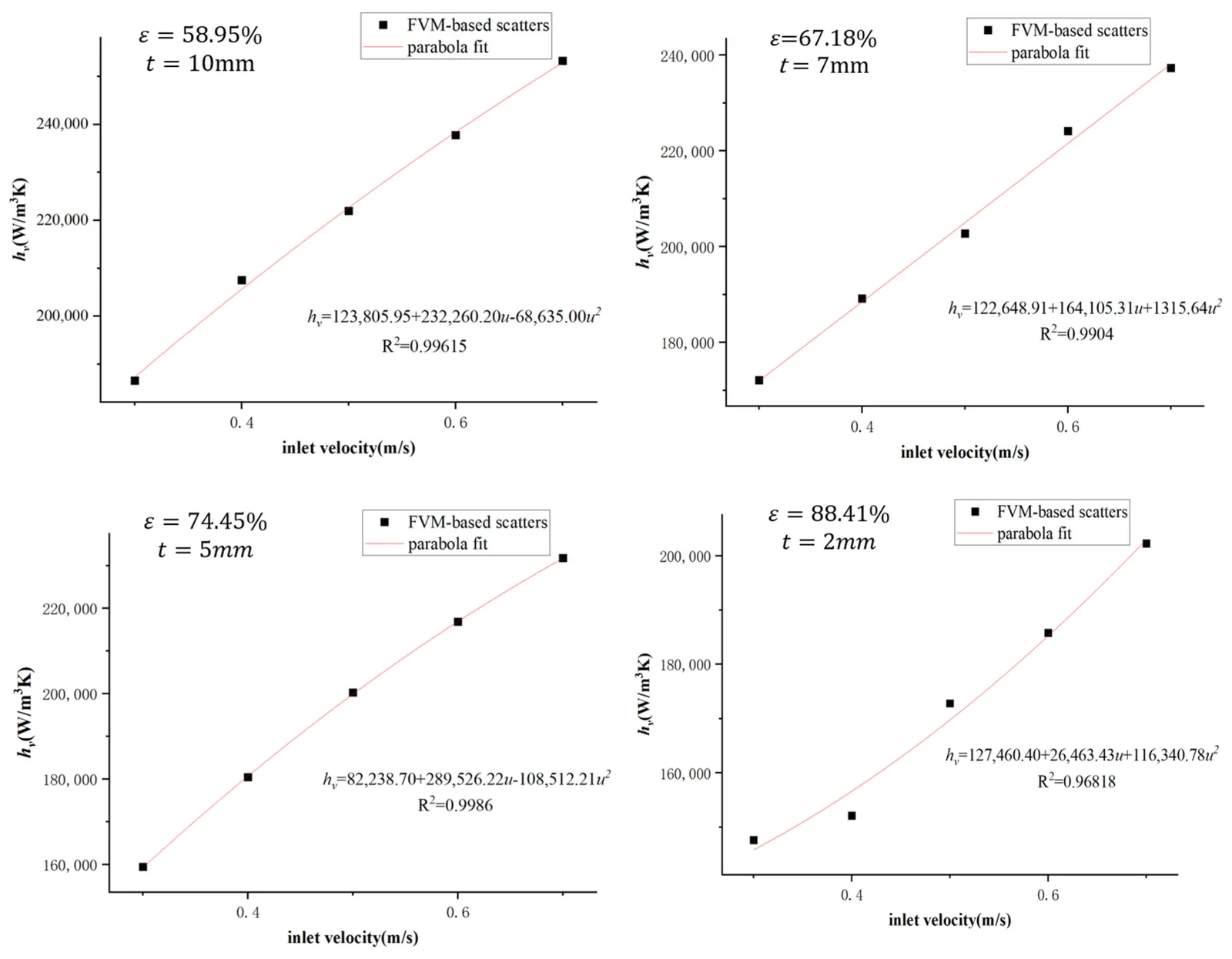
Fitting curve of the volumetric heat transfer coefficient $h_{v}$ with inlet velocity at a specific porosity.

**Figure 10:**
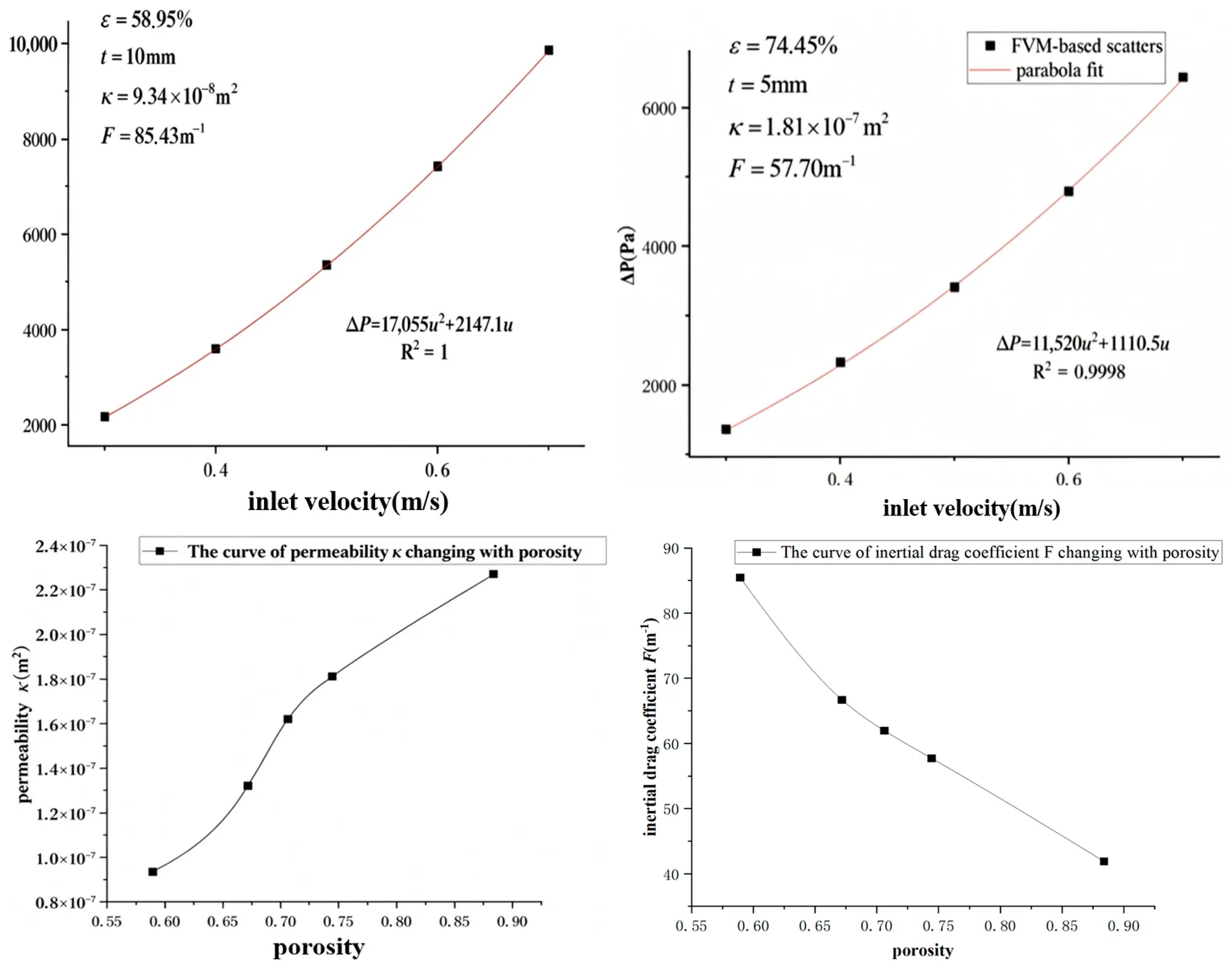
Pressure drop–velocity fits at specific porosity and the curves of permeability and inertial drag coefficient as a function of porosity for Gyroid TPMS structure.

To characterize the flow characteristics within the Gyroid structure, it is essential to establish a corresponding flow effective model, which requires determining two effective parameters: inertial drag coefficient *F* and permeability κ, as shown in Equation (18). By fitting the relationship between the pressure drop and velocity for the Gyroid structures with different porosities, the values of these parameters can be determined, as shown in Figure 9. Figure 10 shows that the fitted curves agree well with the numerical simulation results under various porosity conditions, with correlation coefficients equal to 1. For Gyroid structures with porosities ranging from 58.95% to 88.41%, the inertial drag coefficient *F* and permeability κ of each selected porosity structure are successfully obtained. We have plotted the curves of permeability κ and inertial drag coefficient *F* as a function of porosity, as shown in the following figures.

### 3.3. Results and Discussion

In this study, the equivalent thermal conductivity of the solid phase was set to 42.4 W/(m·K), and that of the fluid phase to 0.446426 W/(m·K). The equivalent volumetric heat transfer coefficient was set to 188,910.83 W/(m^3^·K). Based on these parameters, a temperature-field prediction program was developed in MATLAB.

**Figure 11:**
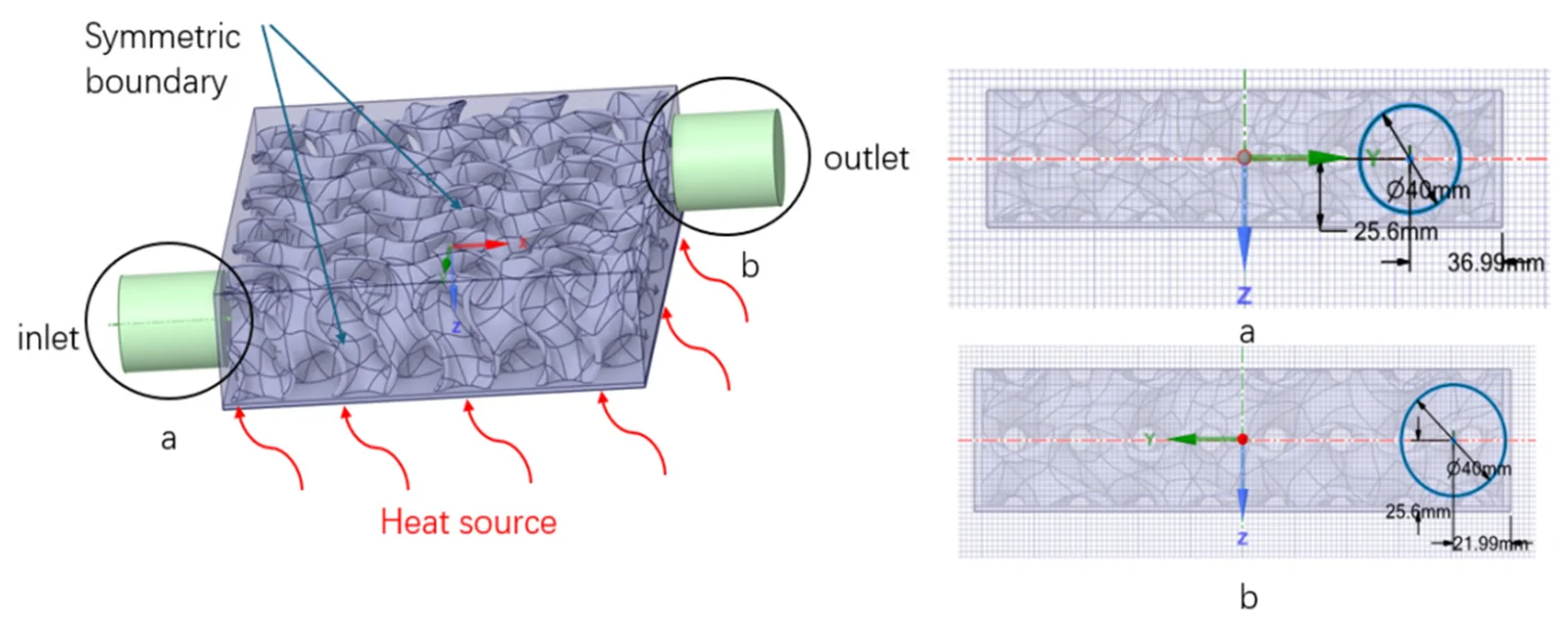
Schematic of the 4 × 4 Gyroid computational model.

First, the model was applied to compute the temperature-field distribution for an arbitrary arrangement of 4 × 4 cells, as shown in Figure 11. The fluid and solid temperatures along the y = x curve and along the y-axis (or x-axis) on the horizontal mid-plane are extracted and compared with the finite volume method (FVM) results obtained from Fluent. Good agreement is achieved between the two approaches. Possible sources of error and their underlying causes were also discussed.

Then, the temperature-field distributions of the dual-medium model were evaluated using arbitrary configurations of 6 × 6 cells and 8 × 8 cells. The comparison method is the same as that used for filling 4 × 4 cells. The grid size and symmetric boundary conditions are also exactly the same as when filling 4 × 4 cells. The results were again compared with those from the FVM in Fluent, confirming that the proposed model achieves good consistency with the FVM.

The CFD cases were divided into calibration cases and validation cases. For calibration, κ and *F* were determined from the flow results of a 4 × 4 array, while $h_{v}$ was determined from the heat-transfer results of a separate 4 × 1 array. These parameters were then applied to the equivalent models without further adjustment. To verify, the 4 × 4 temperature-field was compared with the corresponding geometric analytical CFD results to evaluate the $h_{v}$ transferred from the 4 × 1 array. This comparison is independent of the calibration data used for $h_{v}$, but not of the 4 × 4 flow data used for κ and *F*. The 6 × 6 and 8 × 8 arrays, whose CFD results were not used in any parameter calibration, provide independent validation of the model’s predictions for larger array sizes.

#### 3.3.1. Thermal Analysis for Structures Filled with 4 × 4 Gyroid Cells

**Figure 12:**
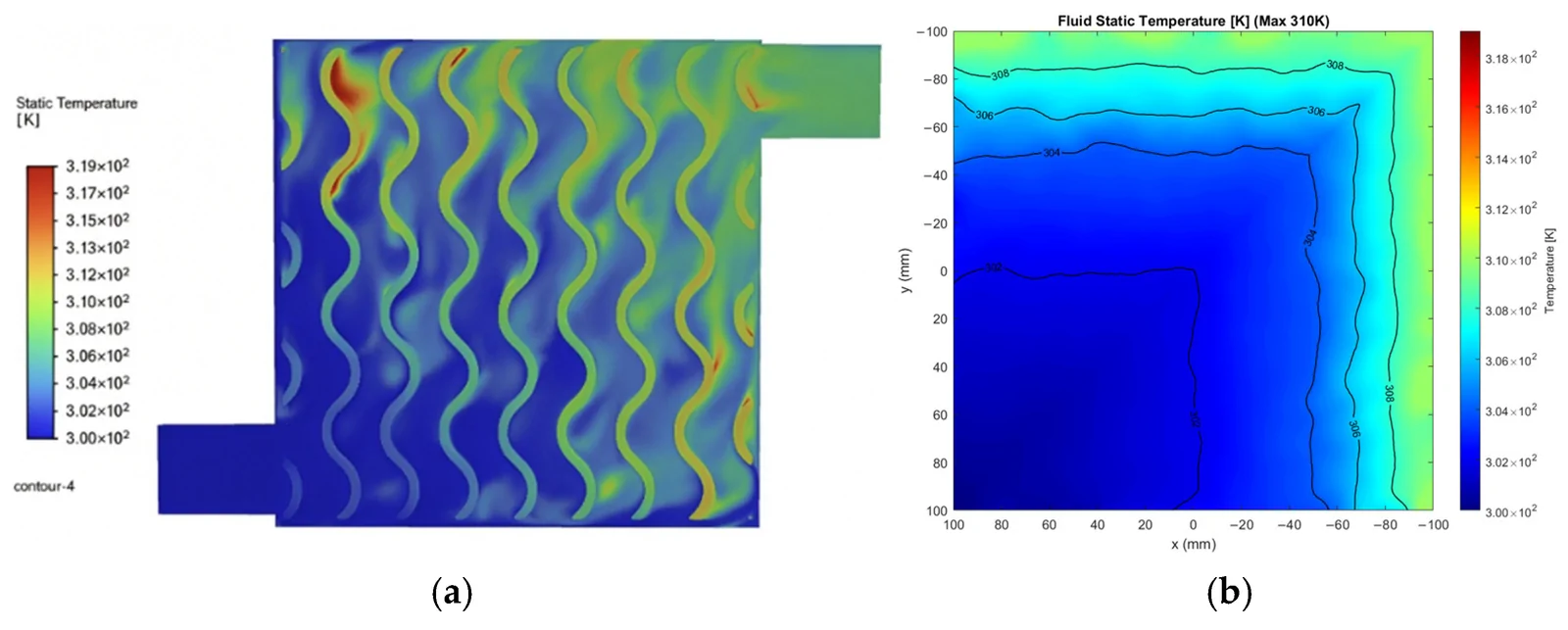
The cross-sectional fluid temperature distribution. (**a**) FVM model fluid temperature contour; (**b**) equivalent model fluid temperature contour.

Figure 11 depicts a 4 × 4 Gyroid structures. Following a comparative thermal analysis, the conclusions are drawn as follows: At the outlet of the FVM model, the fluid temperature is 307 K, but in the equivalent model, the fluid temperature is at 309.5 K, as shown in Figure 12. According to the formula: $Q=\rho_{f}C_{p,f}A_{outlet}v_{outlet}\Delta T$, the heat output per unit time corresponding to each temperature is 644,183.84 J/s and 649,429.64 J/s, respectively, with a relative error of 0.81%.

**Figure 13:**
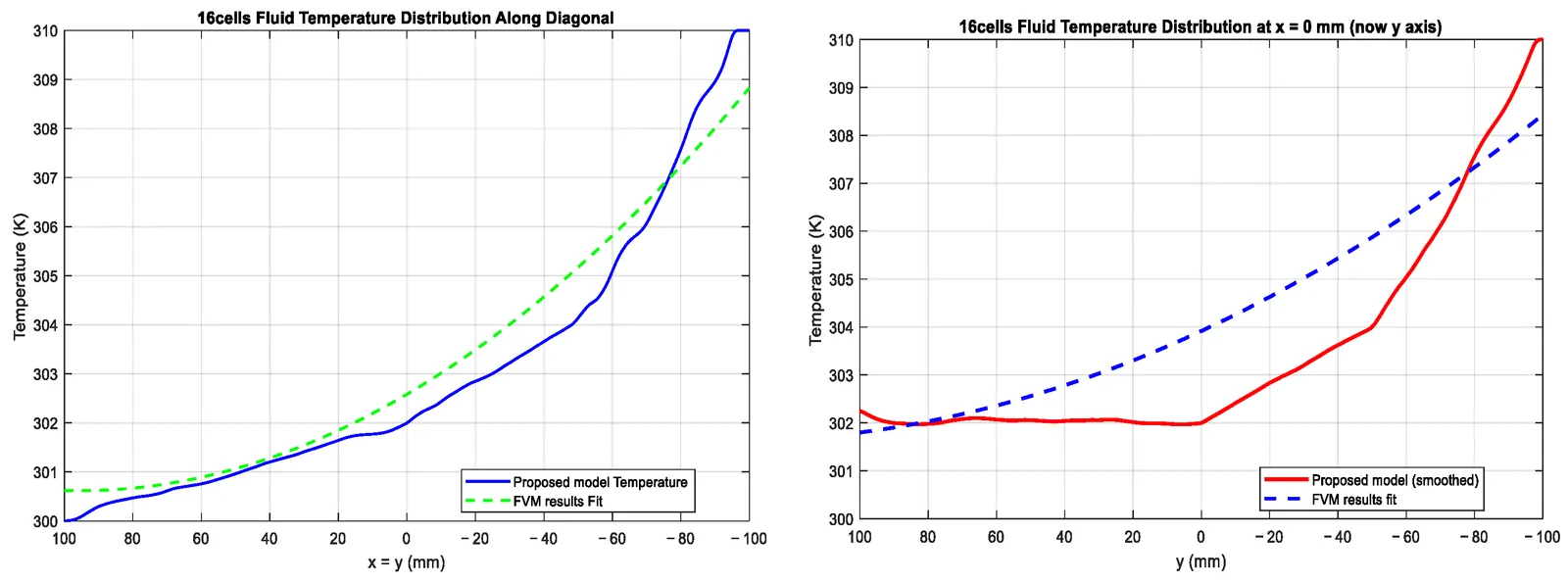
Fluid temperature curve along the diagonal and the y-axis on the mid-plane.

According to the FVM results in Figure 13, on the horizontal mid-plane, the fluid temperature gradually increases from 300.7 K to 309 K along the main diagonal from the bottom left corner to the top right corner. As shown in the left side of Figure 13, when x = y = −49 mm, the maximum deviation between the FVM results and the equivalent model results along the diagonal is 1 K, and the relative error is 1.37%. But in the graph on the right side of the Figure 13, for the temperature curve along the y-axis, the maximum relative error is 2.73%.

**Figure 14:**
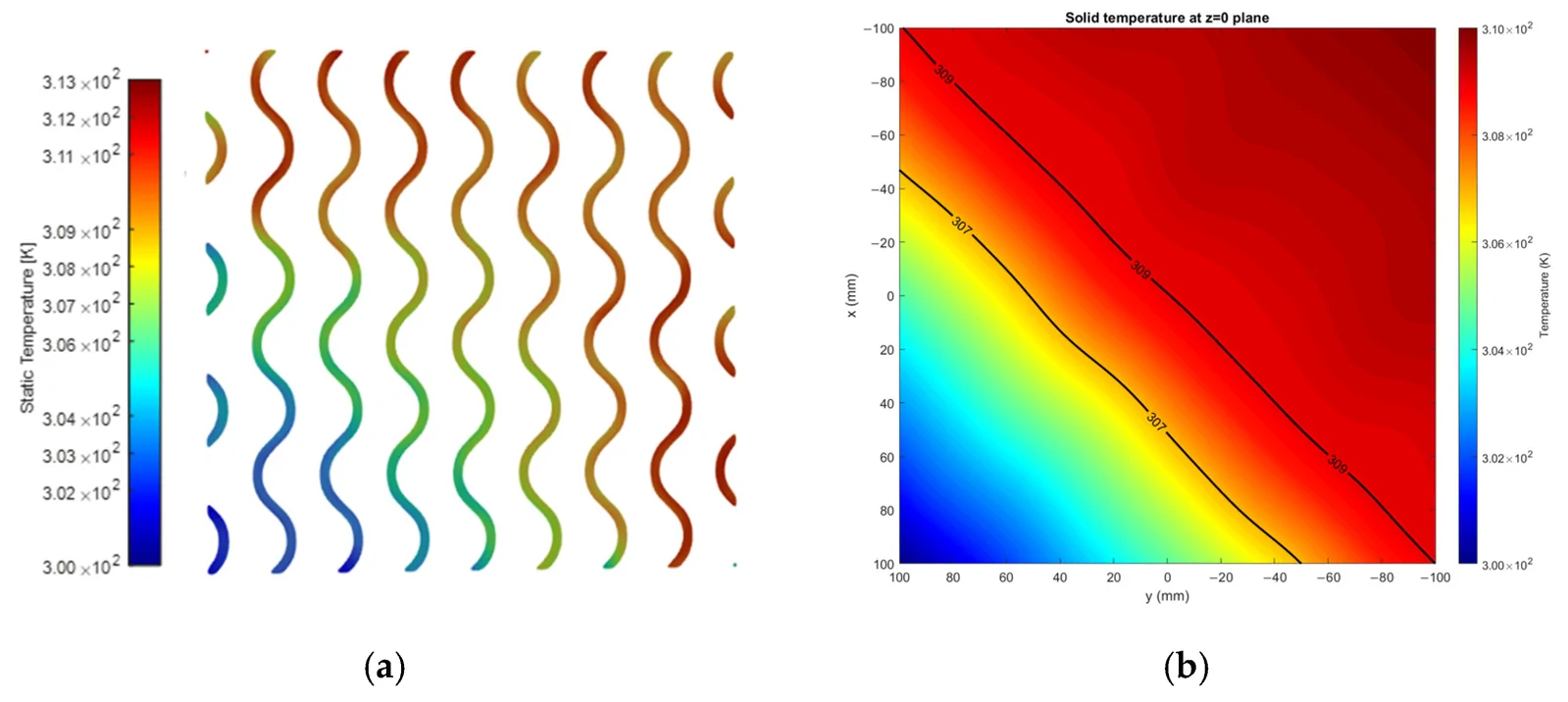
Solid temperature distribution contour on the mid-plane. (**a**) FVM model solid temperature contour; (**b**) equivalent model solid temperature contour.

**Figure 15:**
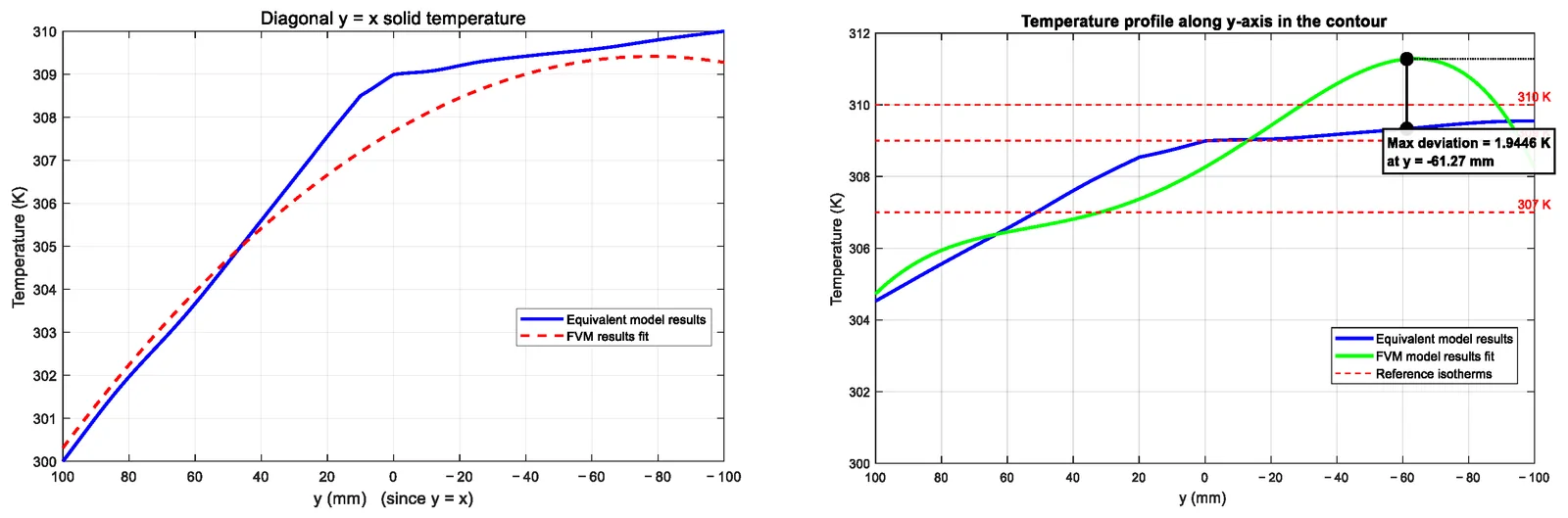
Solid temperature curve along the diagonal and the y axis on the mid-plane.

For solid temperature, we continue to compare the temperature at y = x. From Figure 14 and Figure 15, it can be seen that in the horizontal section of the proposed model, the solid temperature near the inlet is about 300 K, but near the outlet it is about 310 K. Compared with the FVM model results, the equivalent model differs from the FVM results by 0.2 K near the inlet, with the largest difference of 1.2 K at the midpoint y = x = 0, and a difference of 0.8 K near the outlet. The FVM results show temperature growth in the form of a quadratic curve, but in the equivalent model, the temperature increases in the form of almost three oblique straight lines: from y = x = 100 to y = x = 10, the temperature increases from 300 K to 308.5 K; from y = x = 10 to y = x = 0, the temperature increases slowly to 309 K; finally, from y = x = 10 to y = x = −100, the temperature increases slowly to 310 K. In the right side of Figure 15, which shows the solid temperature contour along the y-axis, the maximum error is 1.9446 K between the FVM model results and the equivalent model results, with an error of 2.66%.

**Figure 16:**
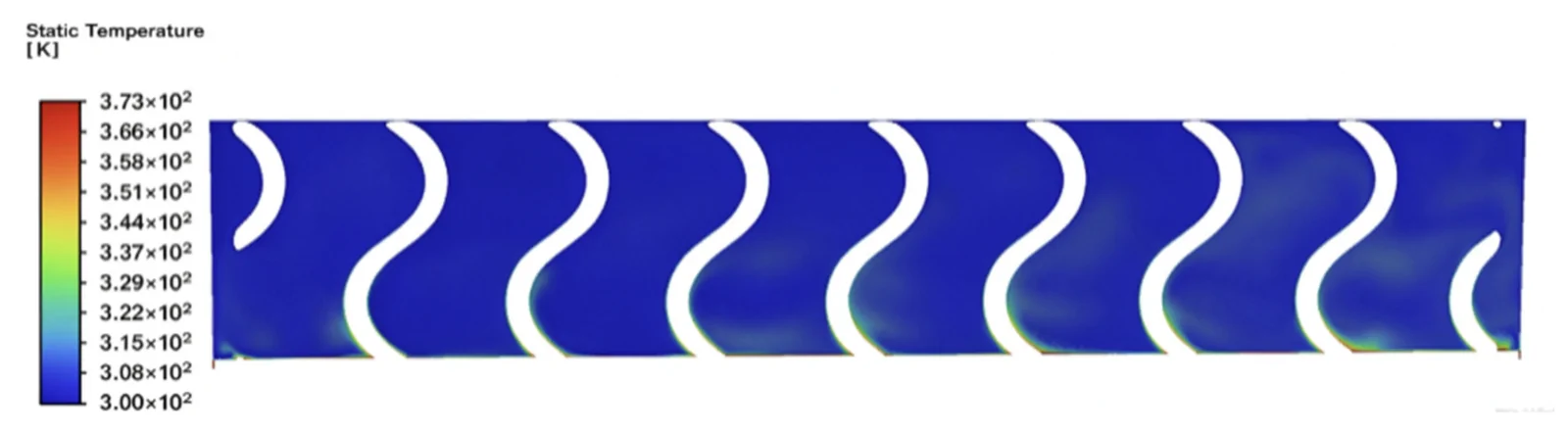
Fluid temperature contour lines on the x = 0 plane in the FVM model.

**Figure 17:**
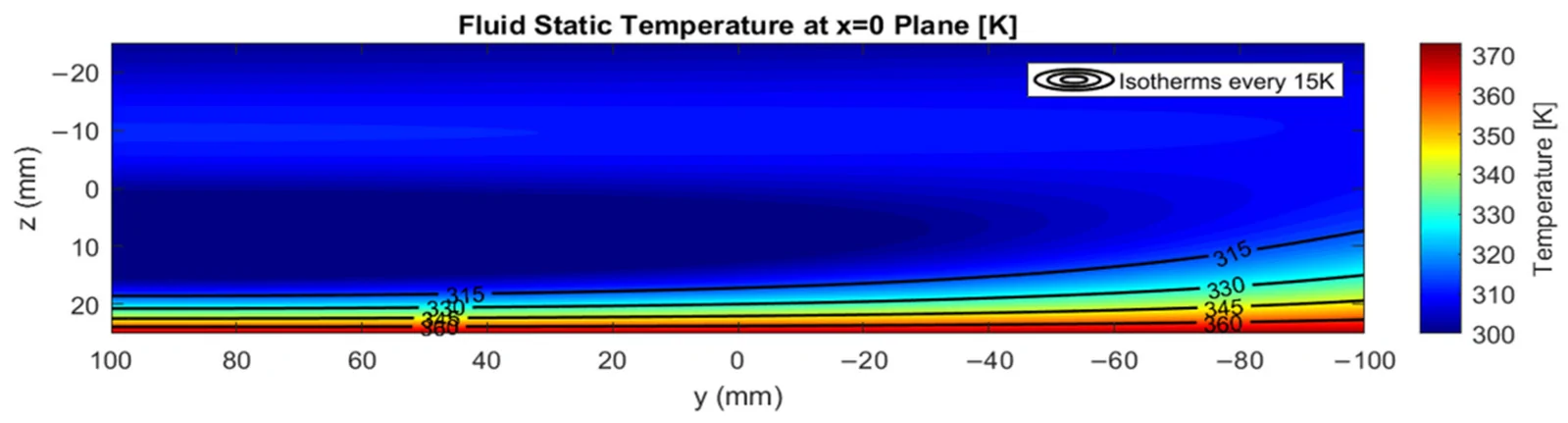
Fluid temperature contour lines on the x = 0 plane in the equivalent model.

**Figure 18:**
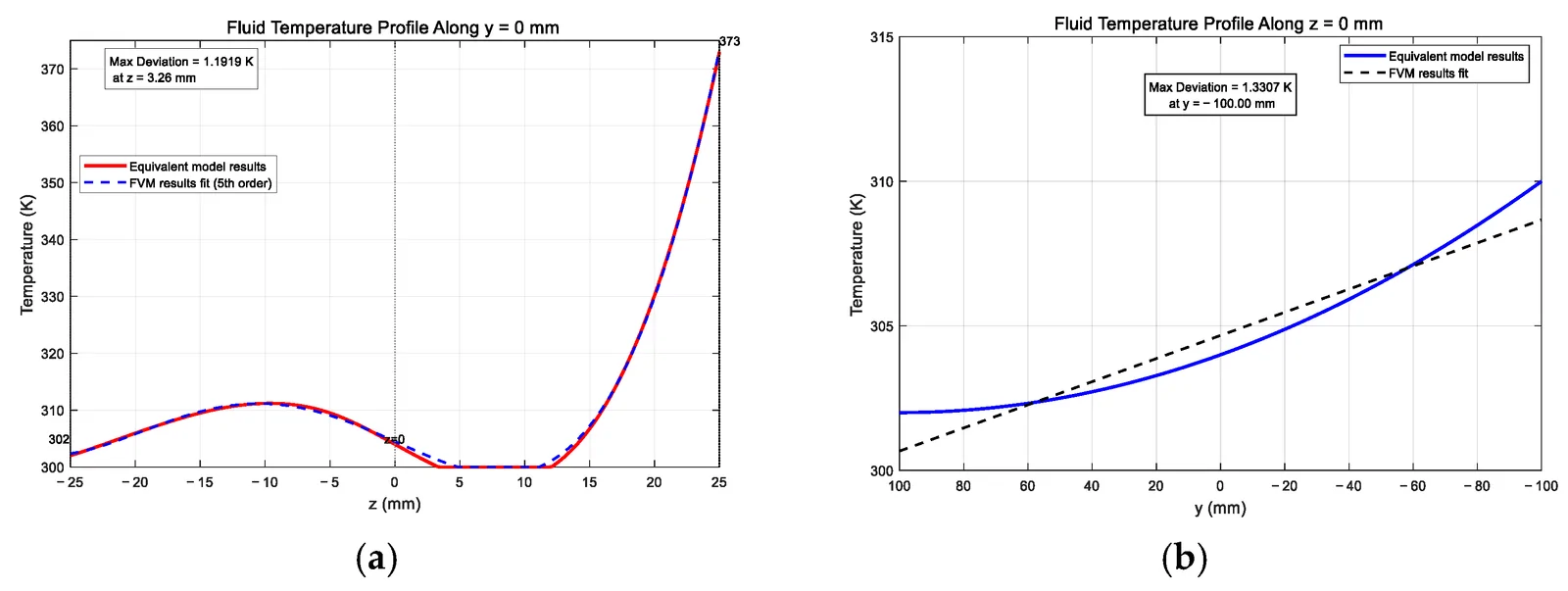
Fluid temperature curves along the horizontal and vertical midlines. (**a**) Fluid temperature along y = 0; (**b**) Fluid temperature along z = 0.

Figure 16 and Figure 17 show the FVM model results and the equivalent model results along the x-axis. The detailed information is shown in Figure 18. Along the vertical centerline y = 0 in Figure 18a, the fluid temperature changes in a nearly 5th-order curve. The FVM model results are in good agreement with the equivalent model results, with a maximum deviation of 1.1919 K occurring at z = 3.26 mm and a relative error of 1.63%. In the fluid temperature curve in Figure 18b along the horizontal centerline z = 0, both the FVM model and the equivalent model grow in the form of quadratic curves, with a maximum deviation of 1.3307 K, occurring at y = −100 mm, and a relative error of 1.82%; from Figure 18, the temperature profiles predicted by the equivalent model along the lines z = 0 and y = 0 are similar to those obtained from the FVM model, supporting the accuracy and effectiveness of the equivalent model.

**Figure 19:**
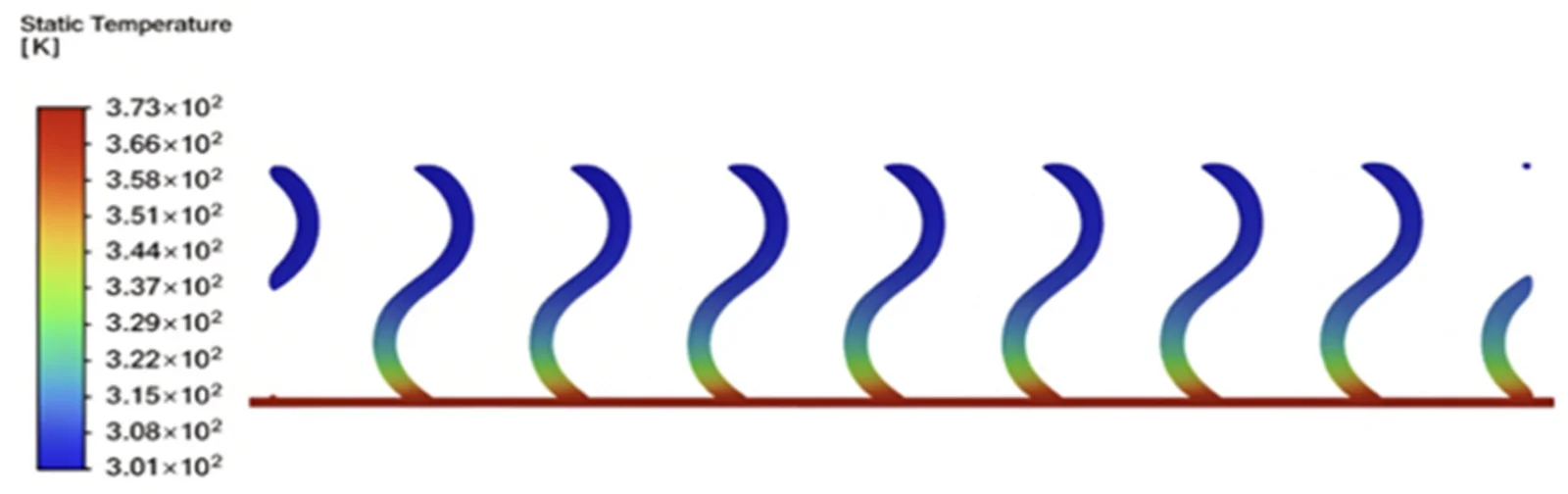
FVM model solid temperature on the y = 0 plane.

**Figure 20:**
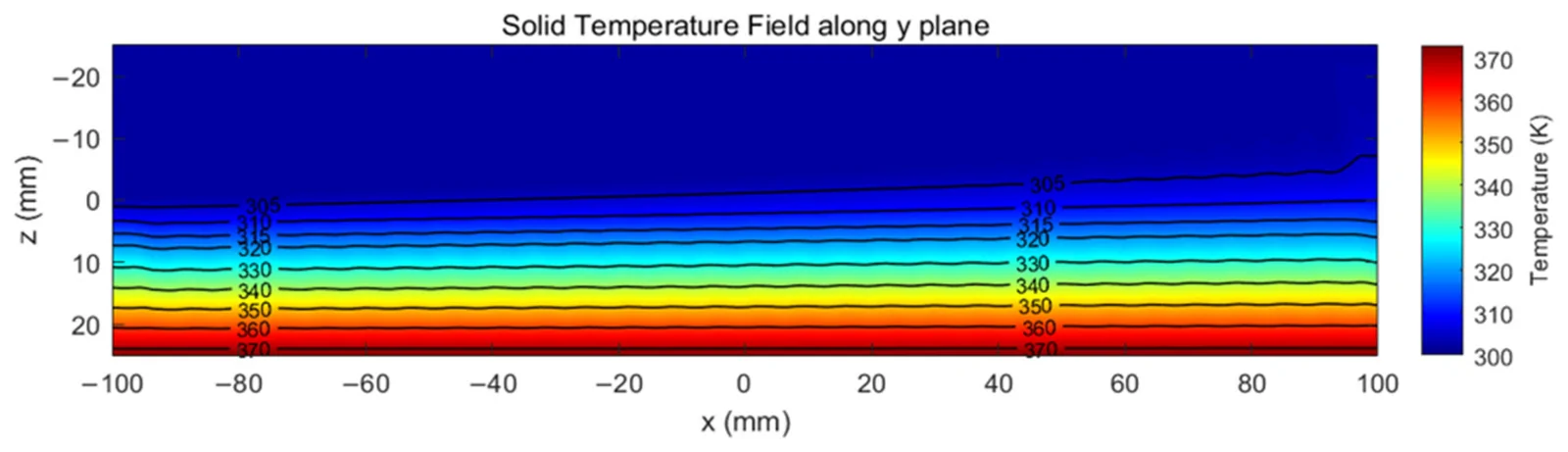
Equivalent model solid temperature on the y = 0 plane.

**Figure 21:**
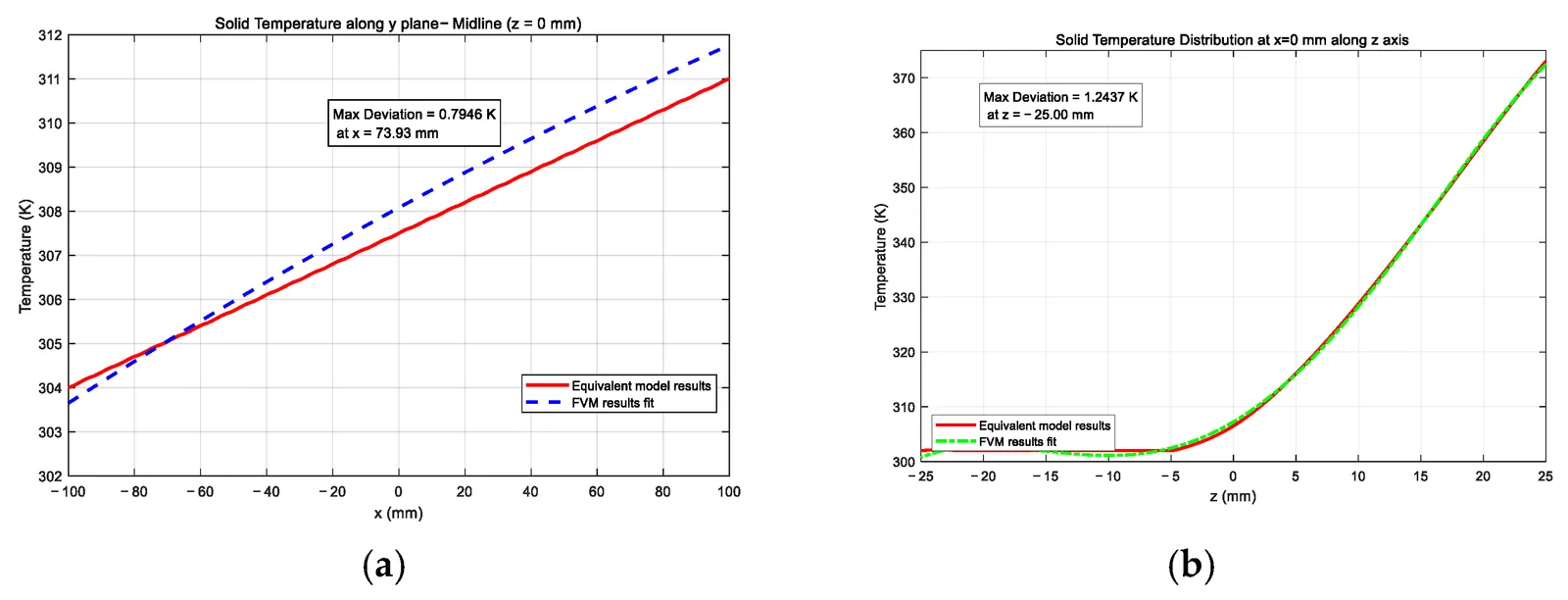
FVM model and equivalent model solid temperature on the y = 0 plane. (**a**) Solid temperature along z = 0; (**b**) solid temperature along x = 0.

As shown in Figure 19 and Figure 20, the difference between the equivalent model and the FVM model is very small. In Figure 21, the solid temperature curves along the horizontal and vertical midlines of the y-plane are depicted in sequence. In Figure 21a, the FVM result curve grows in the form of a quadratic parabola, but in the equivalent model, it grows almost in the form of an oblique straight line, with a maximum deviation of 0.7946 K, occurring at x = 73.93 mm, with a relative error of 1.09%. In Figure 21b both grow in the form of quadratic parabolas, with a maximum deviation of 1.2437 K, occurring at z = −25 mm, and a relative error of 1.70%.

For the 4 × 4 model, the proposed method employs approximately 2,000,000 mesh cells, whereas the FVM approach requires 3,941,514 cells, reducing memory consumption by nearly half. We performed the numerical simulations of this model on a newer laptop with an Intel core ultra 9 275HX CPU, a GEFORCE RTX 5090 graphics card, 64 GB of memory, and a 2 TB hard drive. We used parallel computing. The computational time of the proposed method was 1 min, compared to 20 min for the FVM, achieving a significant reduction in simulation time.

#### 3.3.2. Thermal Analysis for Structures Filled with 6 × 6 Gyroid Cells

**Figure 22:**
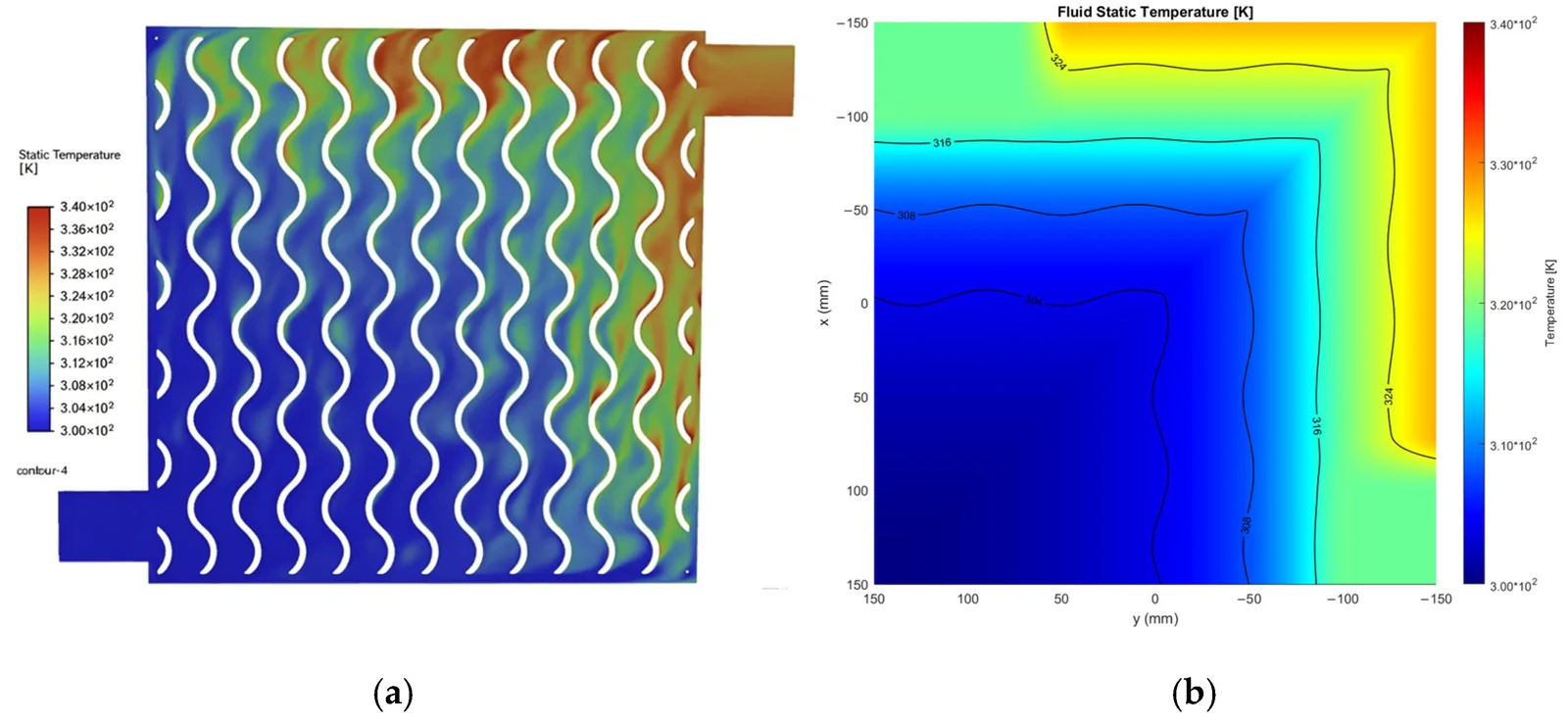
Temperature distribution contour of the fluid phase on the horizontal mid-cross section. (**a**) FVM model z = 0 plane fluid temperature; (**b**) equivalent model z = 0 plane fluid temperature.

From the comparison of Figure 22a,b, it can be seen that the lowest temperature of the fluid phase in the FVM model occurs at the inlet at 300 K, and the highest temperature occurs near the outlet, at 328 K. However, in the equivalent model, the highest temperature is 330.2 K, and the FVM model corresponding to the outlet temperature and the derived heat is 688,248.53 J/s, while the corresponding heat derived from the equivalent model is 692,864.83 J/s, with a relative error of 0.67%.

**Figure 23:**
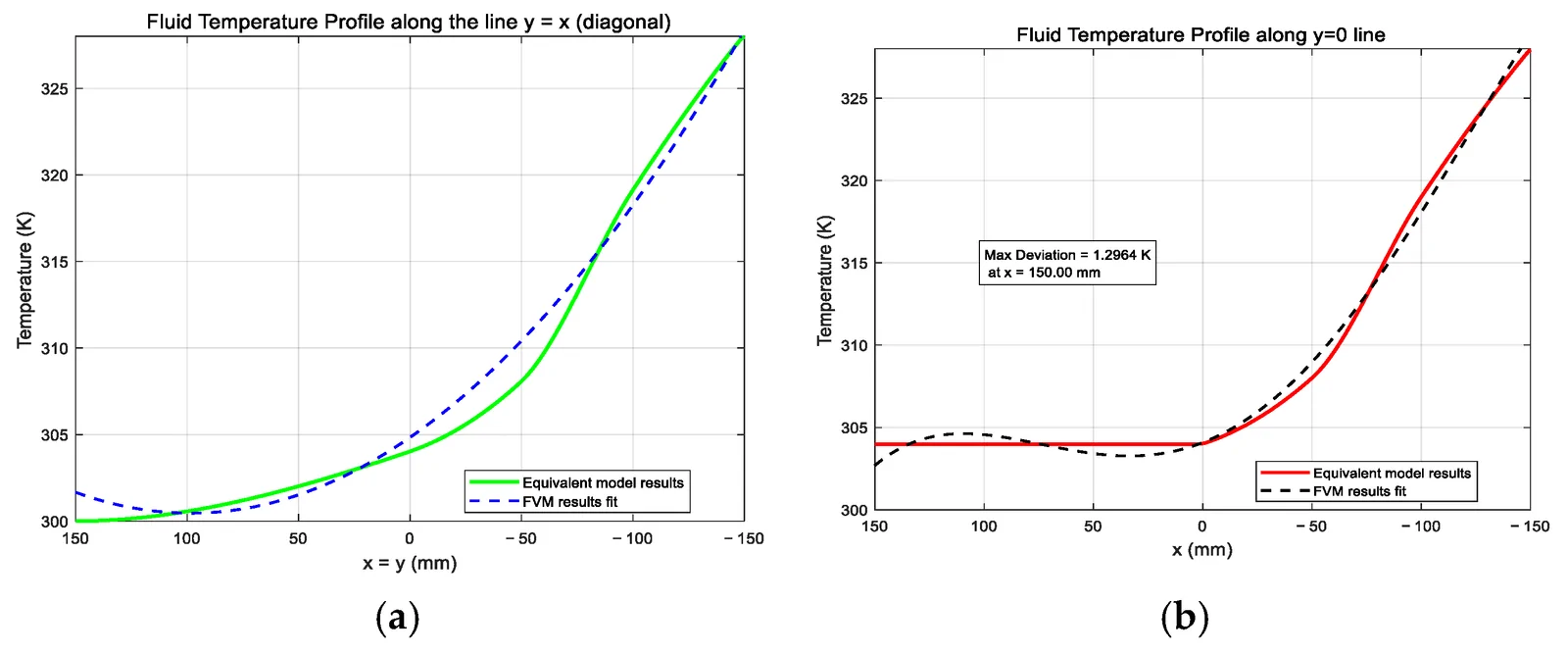
Temperature distribution curves of the fluid phase along the diagonal and the x-axis. (**a**) Fluid temperature along y = x; (**b**) fluid temperature along y = 0.

In Figure 23a, for the temperature curve along the y = x line, the maximum deviation occurs at x = −50 mm, with a deviation value of 2 K and a relative error of 2.73%. Both curves in these two groups grow in the form of parabolas. In Figure 23b, the maximum deviation between the equivalent model and the FVM model along the y = 0 line occurs at x = 150 mm, with a deviation value of 1.2964 K and a relative error of 1.77%.

**Figure 24:**
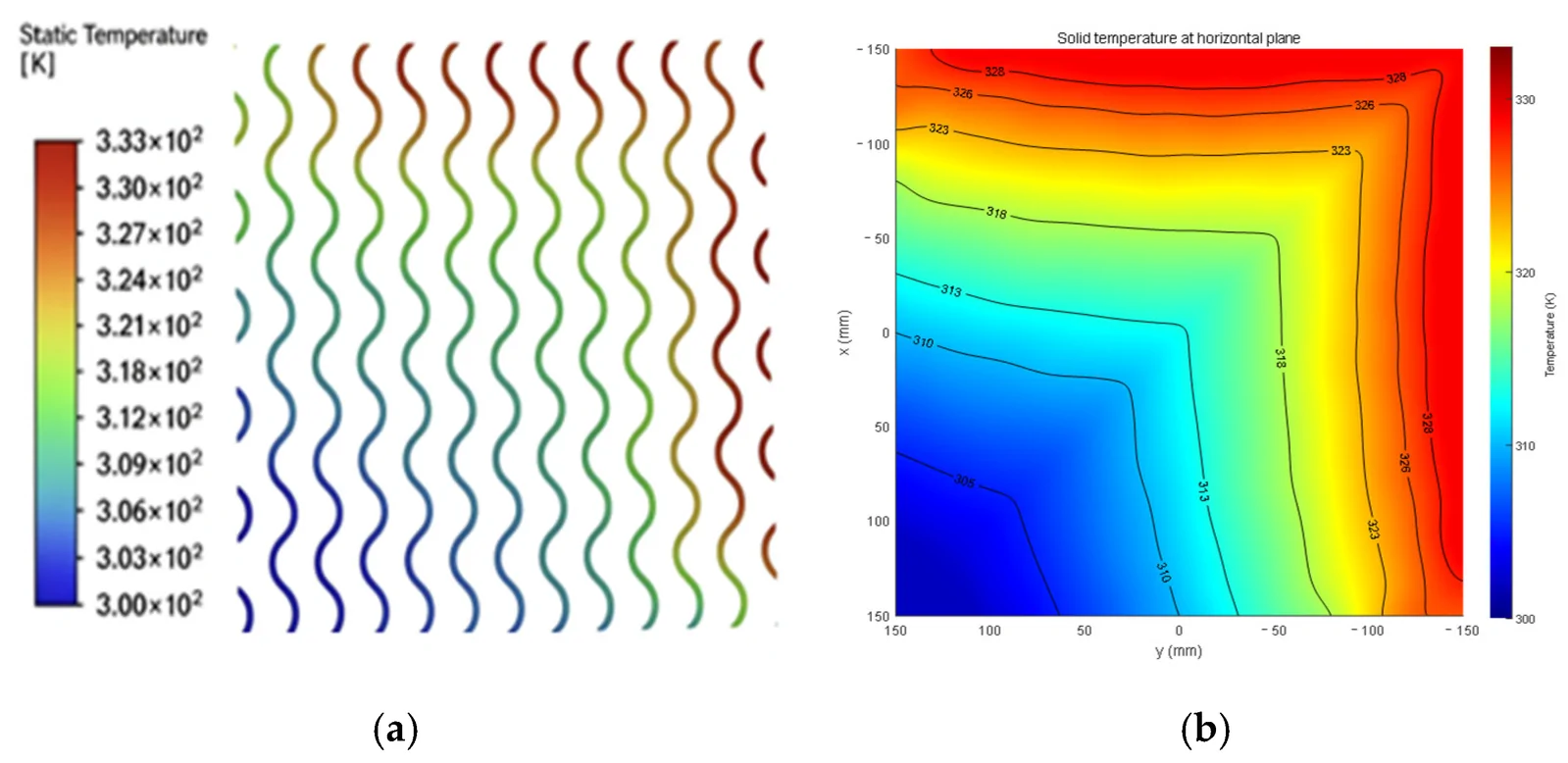
Solid temperature distribution contour on the mid-plane. (**a**) FVM model solid temperature contour on the horizontal mid-plane; (**b**) equivalent model solid temperature contour on the horizontal mid-plane.

**Figure 25:**
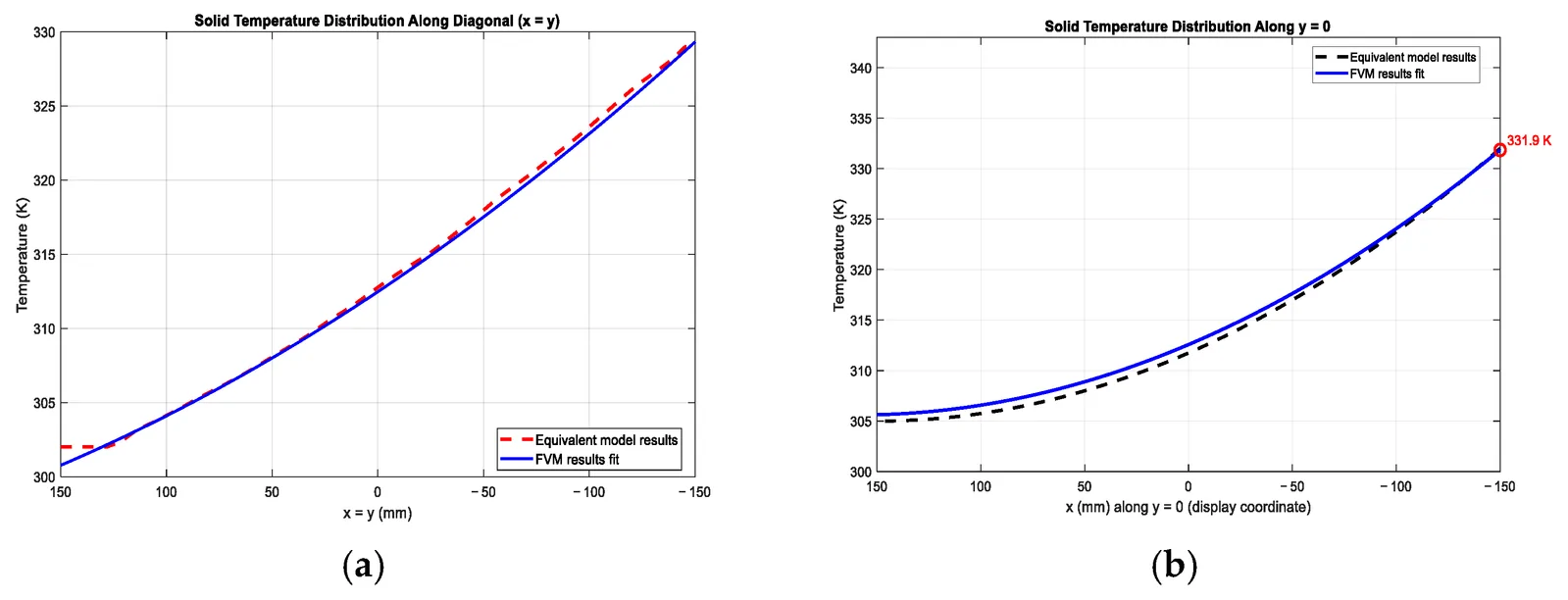
Mid-surface solid temperature curve along the diagonal and y = 0. (**a**) Solid temperature along the diagonal; (**b**) solid temperature along y = 0.

As shown in Figure 24, the error between the equivalent model and the FVM model mainly occurs near the two intersection points of the sub-diagonal and the computational domain, but the error is relatively small in other parts. According to Figure 25, the maximum error along the diagonal temperature curve is 1.68%. Both curves grow almost entirely in the form of quadratic functions. The curve along the diagonal has a minimum temperature of 301 K and a maximum temperature of 329 K. The curve along y = 0 has a minimum temperature of 305 K and a maximum temperature of 331.9 K, and the maximum error along the y = 0 temperature curve is 0.86%. The equivalent model results are in good agreement with the FVM model results.

**Figure 26:**
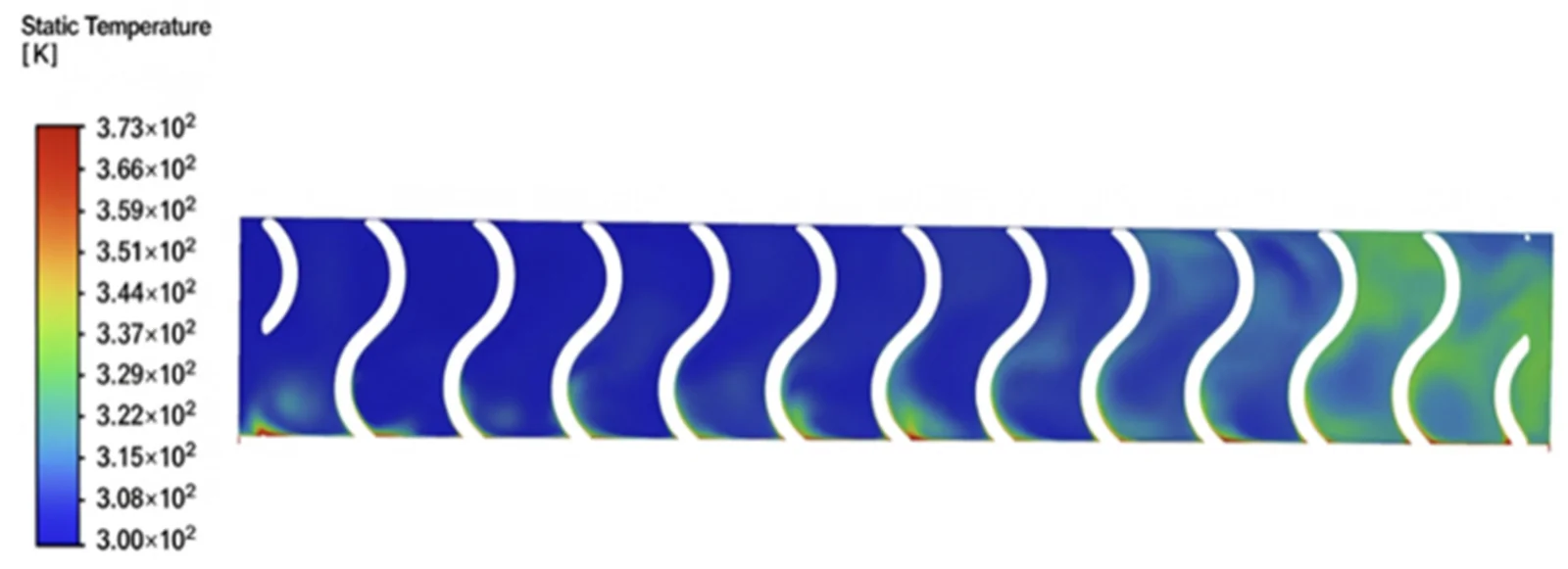
FVM model fluid temperature contour on the y = 0 plane.

**Figure 27:**
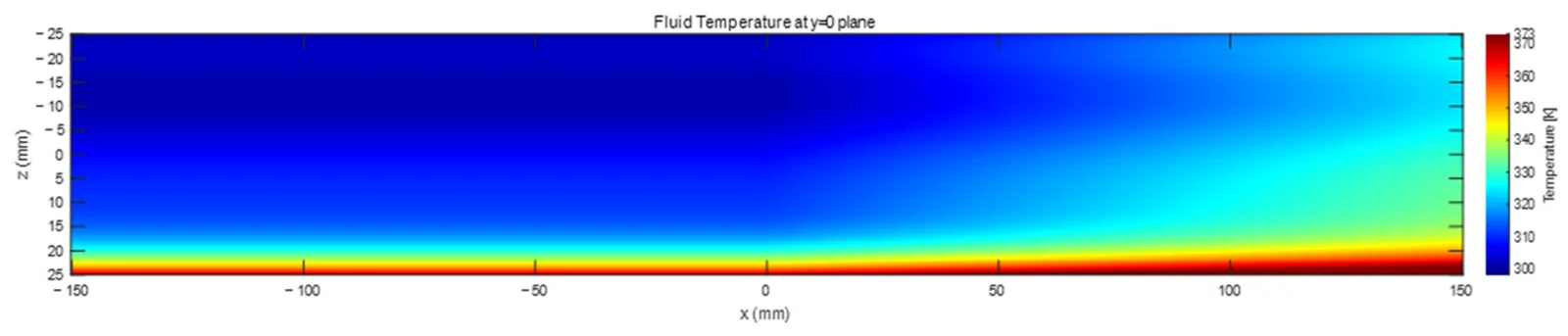
Equivalent model fluid temperature contour on the y = 0 plane.

**Figure 28:**
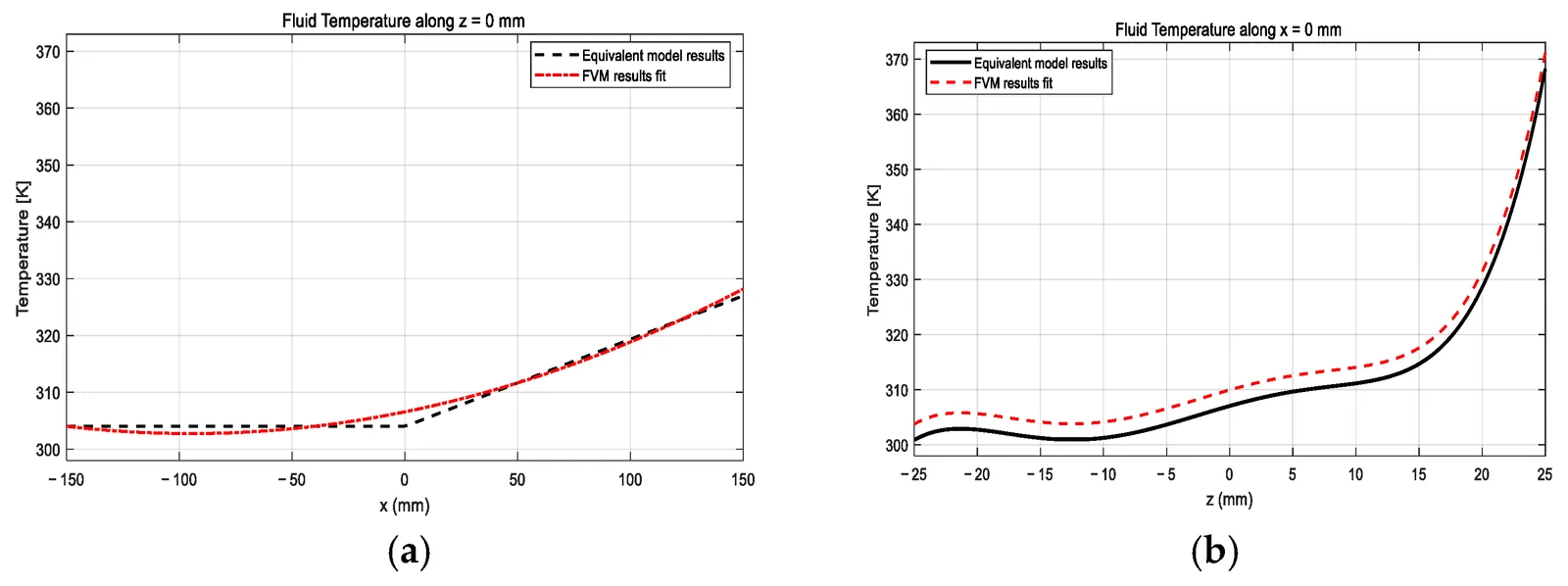
Fluid temperature curve along z = 0 and x = 0. (**a**) Fluid temperature along z = 0; (**b**) Fluid temperature along x = 0.

From the comparison of Figure 26 and Figure 27, it can be seen that the equivalent model has a small error at x = 130 mm–150 mm, while other positions are in good agreement with the FVM model. According to Figure 28, in the curve along z = 0 direction, the maximum error is 3.30%. The equivalent model increases in the form of two oblique straight lines, while the FVM model increases in the form of a quadratic curve; in the graph along x = 0, both model curves increase in the form of a 5th-order curve. The relative error is 3.77%.

**Figure 29:**
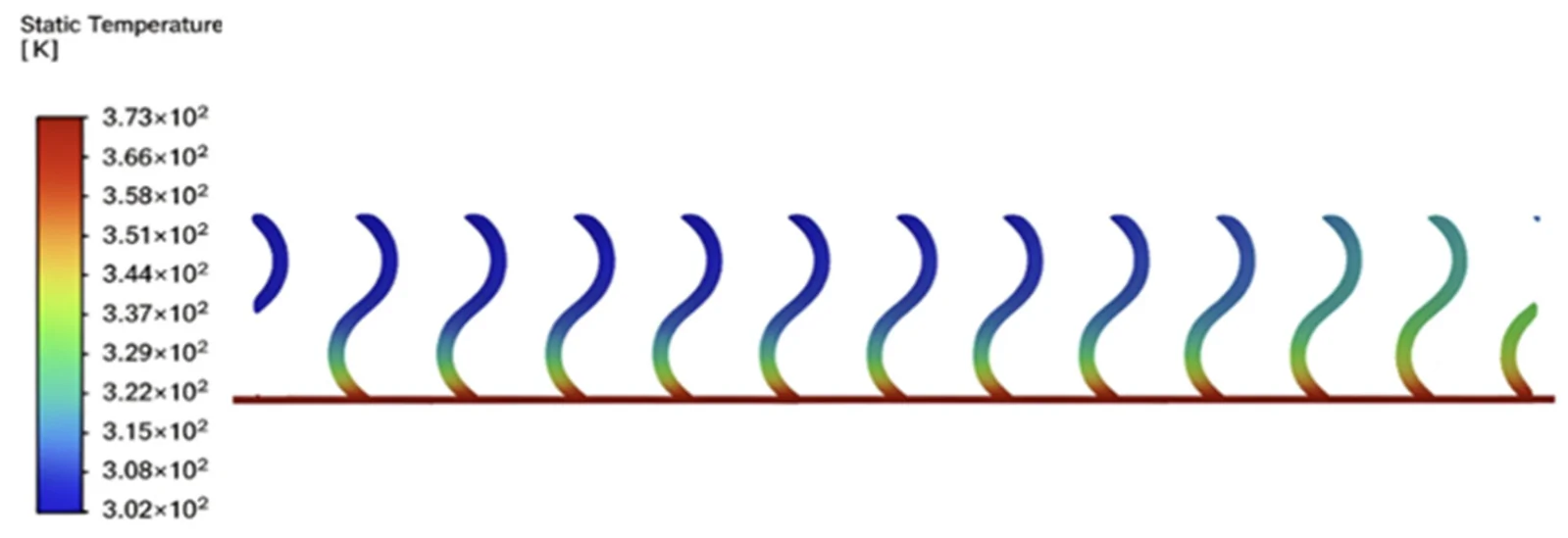
Solid temperature contour of the FVM model on the y = 0 plane.

**Figure 30:**
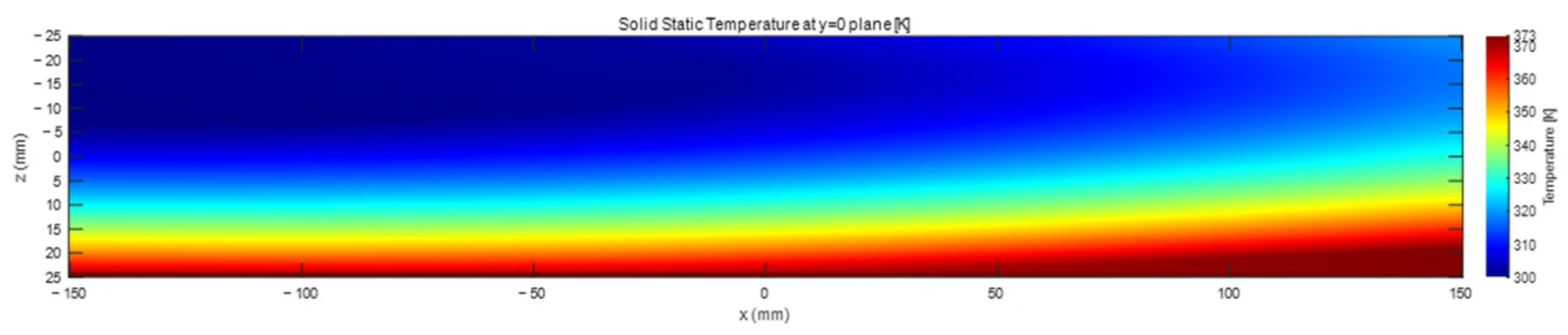
Solid temperature contour of the equivalent model on the y = 0 plane.

**Figure 31:**
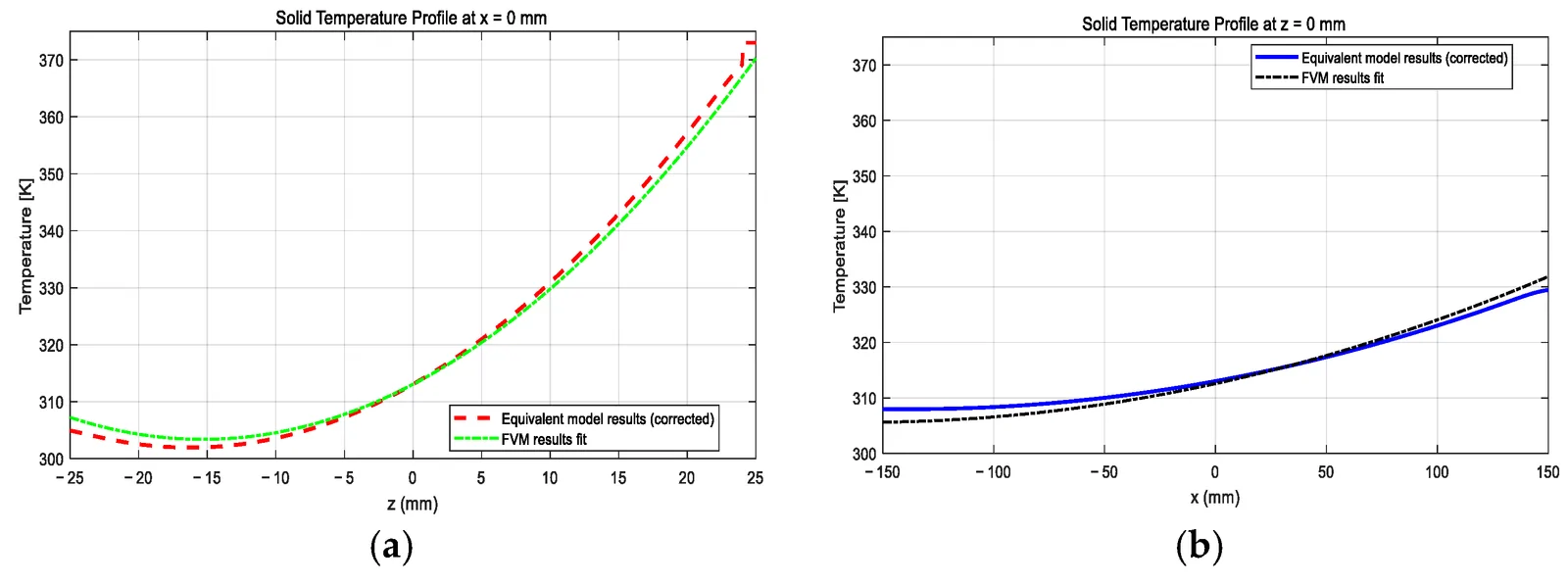
Solid temperature curve on the y = 0 plane. (**a**) Solid temperature curve along x = 0; (**b**) solid temperature curve along z = 0.

From the comparison between Figure 29 and Figure 30, it can be seen that the error of the equivalent model mainly comes from the interval x = 130 mm–150 mm, while in other places, the error is extremely small. According to Figure 31, the maximum relative error between the FVM model and the equivalent model along the temperature curve with x = 0 is 3.16%; the maximum relative error between the two models along the temperature curve with z = 0 is 3.23%. The four curves in the above two sets all increase in the form of quadratic curves.

For the 6 × 6 model, the proposed method employs approximately 4,500,000 mesh cells, whereas the FVM approach generates 8,568,294 cells, reducing memory consumption by nearly half. The computational time of the proposed method was 1.5 min, compared to 35 min for the FVM, achieving a significant reduction in simulation time.

#### 3.3.3. Thermal Analysis for Structures Filled with 8 × 8 Gyroid Cells

**Figure 32:**
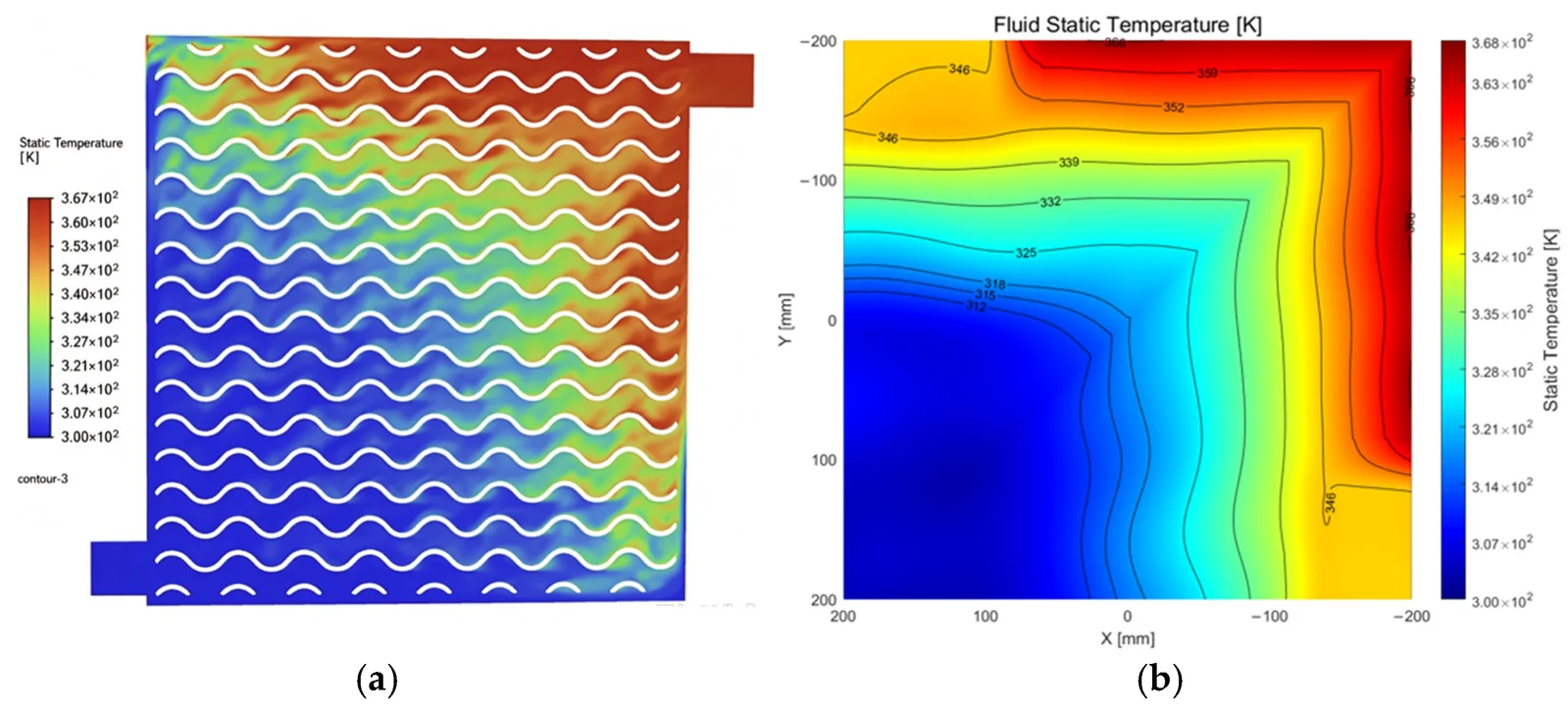
Temperature distribution contour of the fluid phase on the horizontal mid-cross-section. (**a**) FVM model fluid temperature; (**b**) equivalent model fluid temperature.

Comparing the two figures (a) and (b) in Figure 32, it can be seen that the error mainly exists at the two intersection points of the sub-diagonal and the computational domain, while at other positions, the error is relatively small. As shown in Figure 32a, the outlet temperature of the FVM model is 362.5 K; as shown in Figure 32b, the outlet temperature of the equivalent model is 364.8 K; the heat output per unit time corresponding to the outlet temperature is 760,640.53 J/s and 765,466.66 J/s; the relative error is 0.63%.

**Figure 33:**
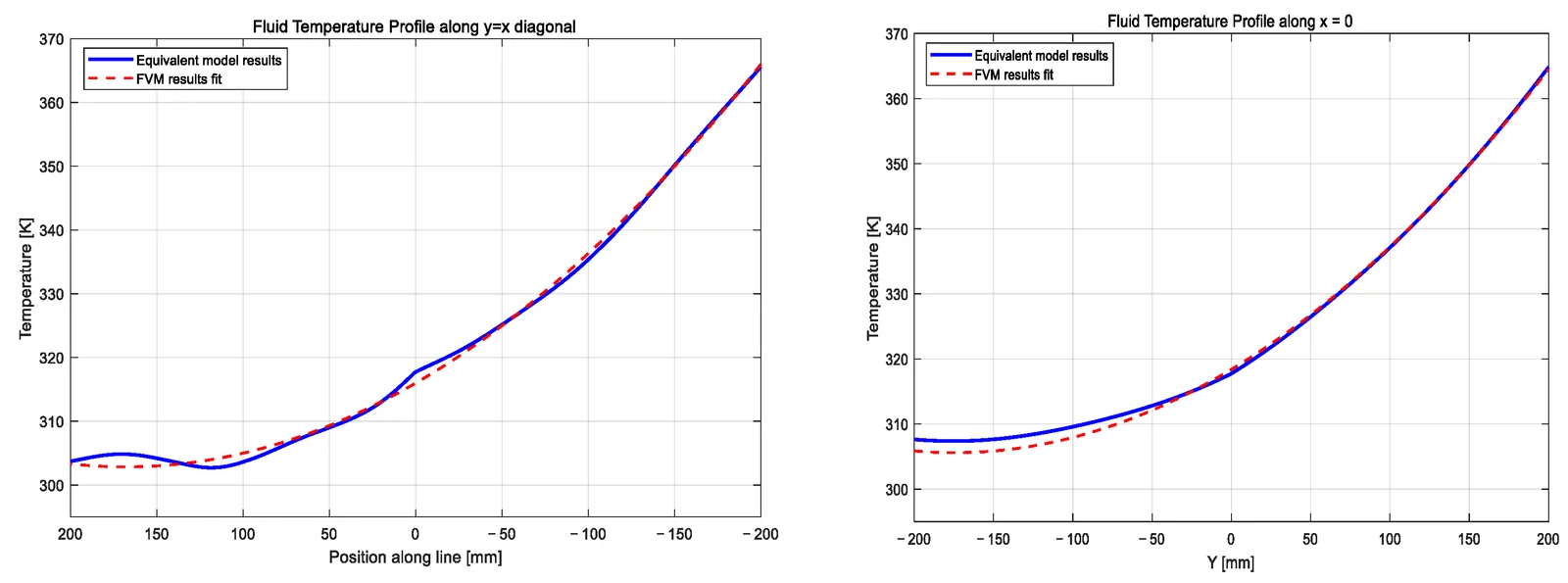
Temperature distribution curves of the fluid phase along the diagonal and y-axis.

In Figure 33, the maximum relative error of the equivalent model along the temperature curve of y = x compared to the FVM results is 2.72%. In the temperature curve graph with x = 0 (y-axis), the maximum relative error is 2.45%. The temperature curve of the equivalent model is in good agreement with that of the FVM model, which proves the accuracy and effectiveness of the equivalent model.

**Figure 34:**
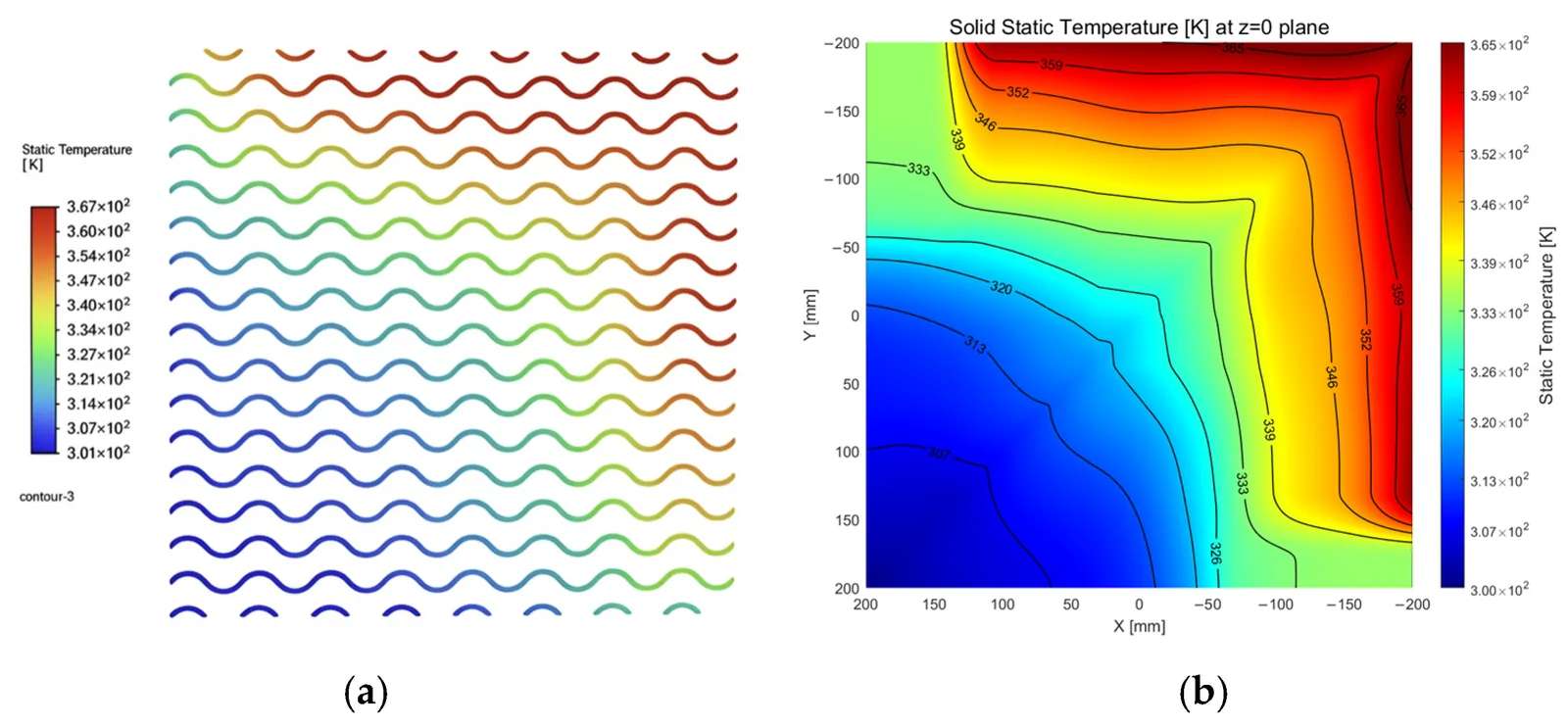
Solid temperature distribution contour on the horizontal mid-surface. (**a**) Solid temperature contour of FVM model; (**b**) Solid temperature contour of equivalent model.

Figure 34 shows the temperature distribution of the solid in the FVM model and the equivalent model along the horizontal mid-plane.

**Figure 35:**
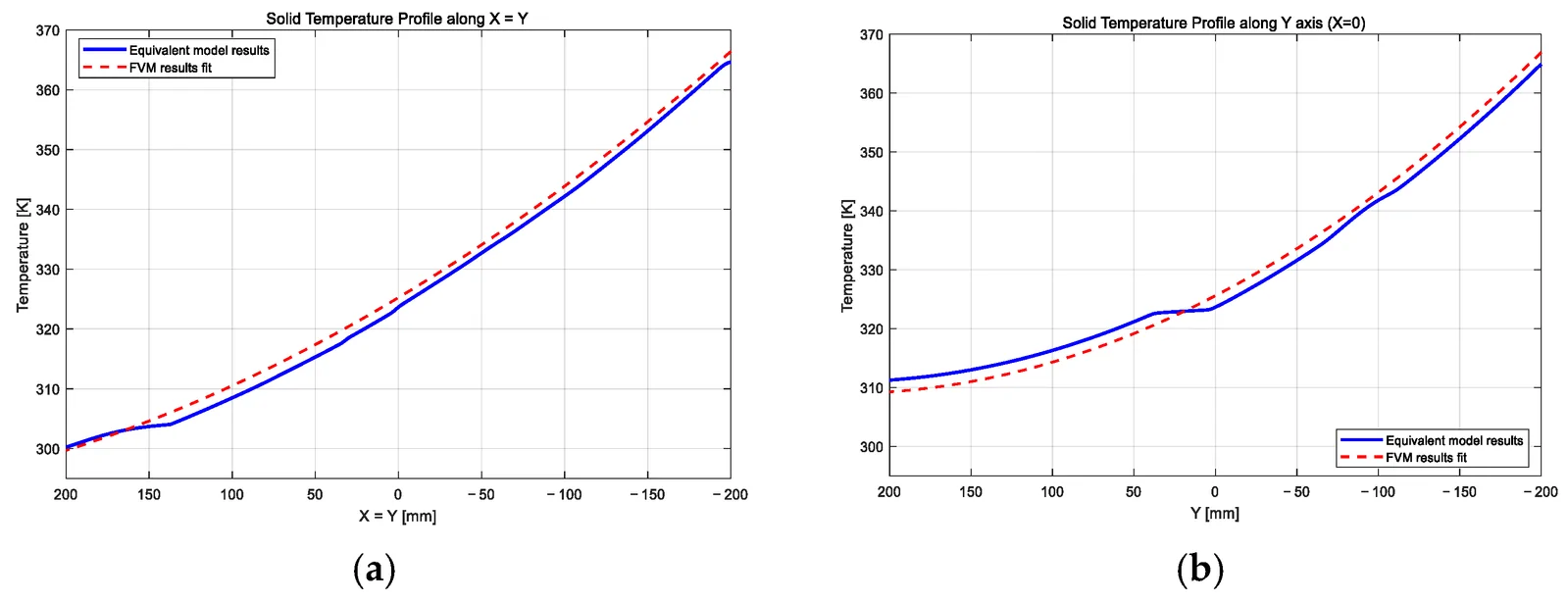
Solid temperature curve at z = 0 plane. (**a**) Along diagonal solid temperature; (**b**) Along y-axis solid temperature.

From the comparison of Figure 35a,b, it can be seen that the error between the equivalent model and the FVM model is very small. In Figure 35a along the temperature curve of y = x, the maximum error between the FVM model results and the equivalent model results is 2.94%, and the maximum deviation is 2.149 K. While in Figure 35b, the maximum error between the temperature curves along the y-axis is 2.74%, and the maximum deviation is 2.001 K. This error is within an acceptable range.

**Figure 36:**
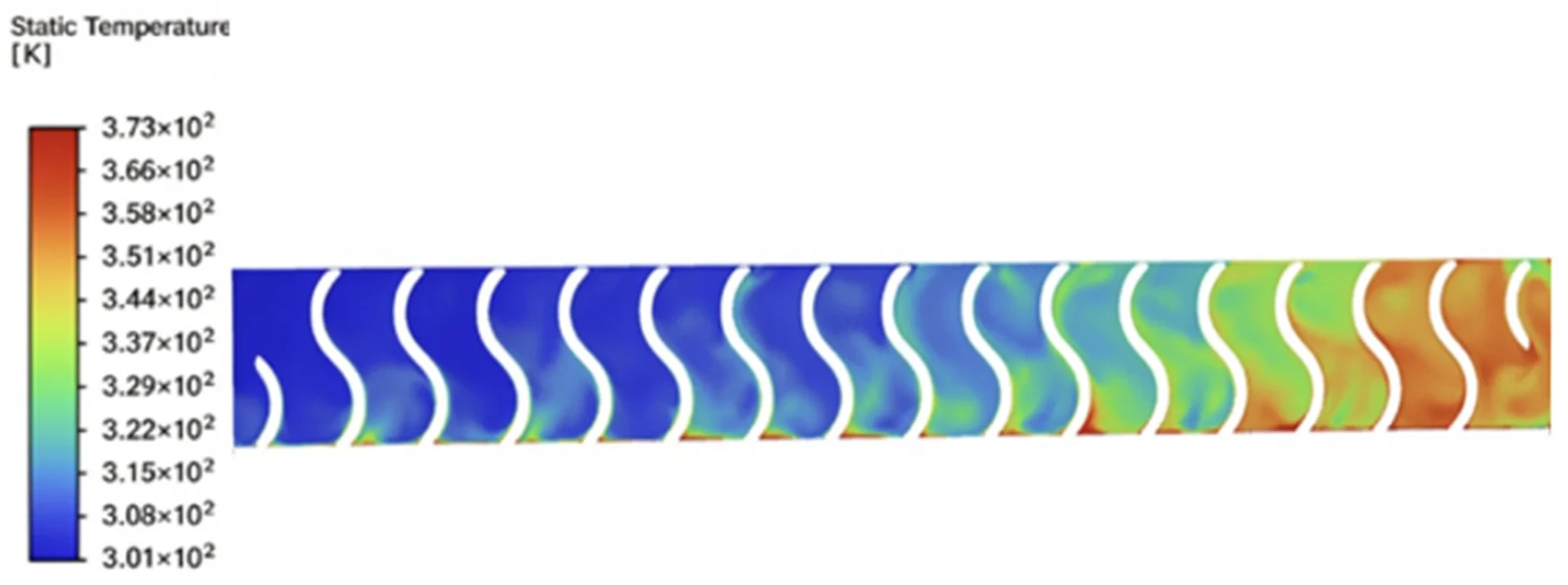
Fluid temperature contour of FVM model on the y = 0 plane.

**Figure 37:**
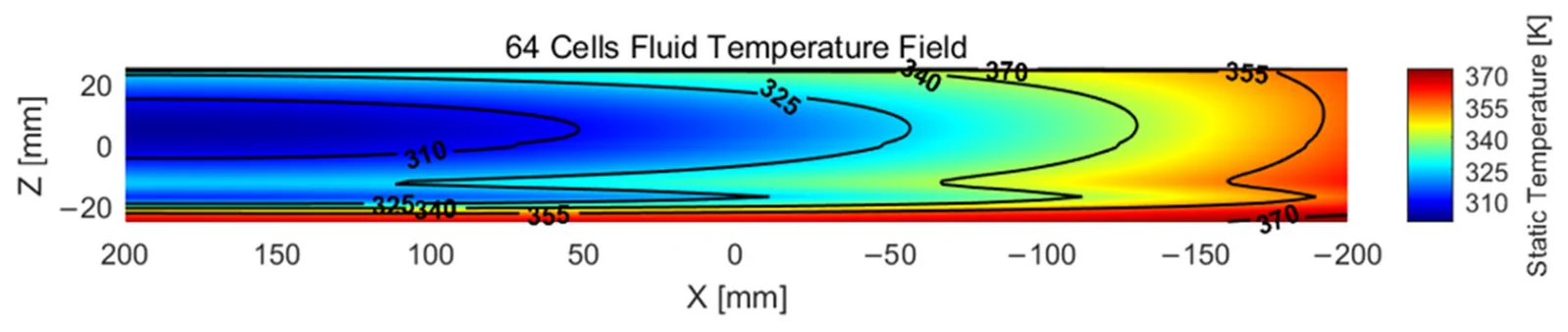
Fluid temperature contour of equivalent model on the y = 0 plane.

**Figure 38:**
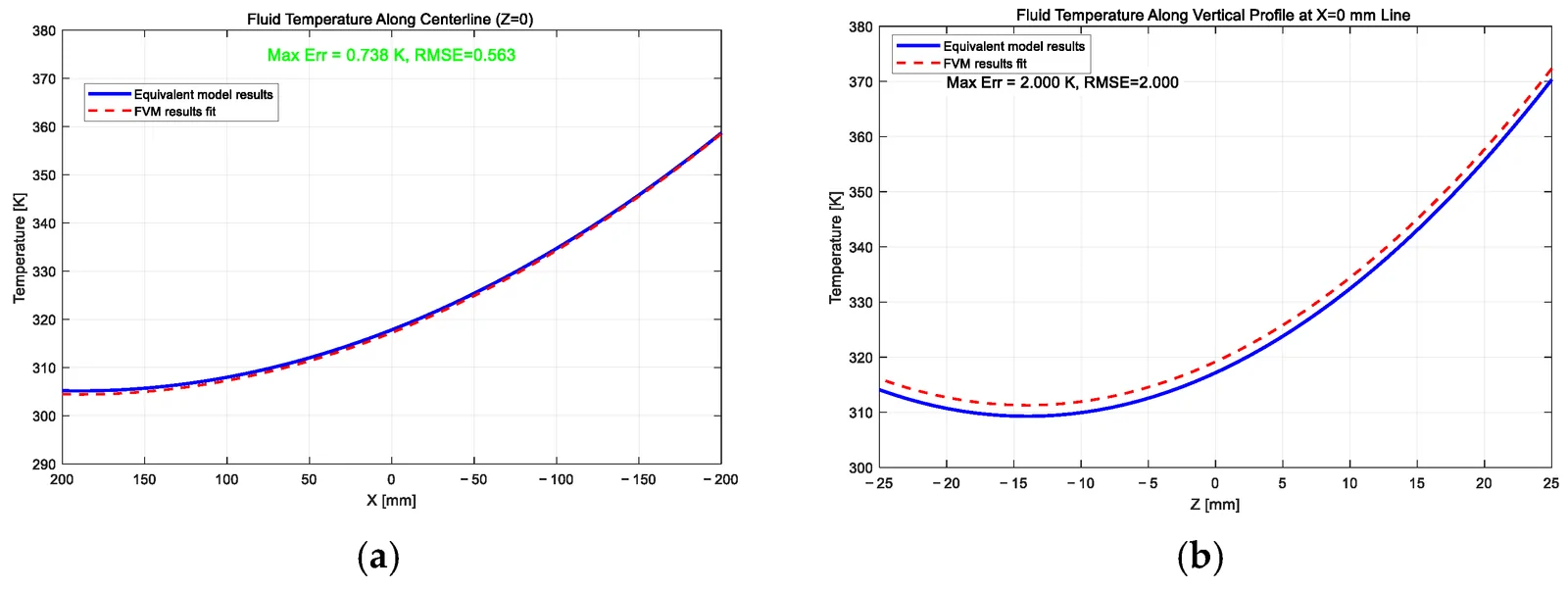
Fluid temperature curve on the y = 0 plane. (**a**) Fluid temperature along z = 0; (**b**) Fluid temperature along x = 0.

From the comparison between Figure 36 and Figure 37, it can be seen that the equivalent model has a certain error compared to the FVM result at x = 0, but the error is very small in other parts. According to Figure 38a, in the temperature curve along z = 0, the maximum error between the equivalent model and the FVM model is 0.738 K, and the relative error is 1.01%. However, in the temperature curve along x = 0 in Figure 38b, the maximum deviation is 2 K, and the relative error is 2.73%.

**Figure 39:**
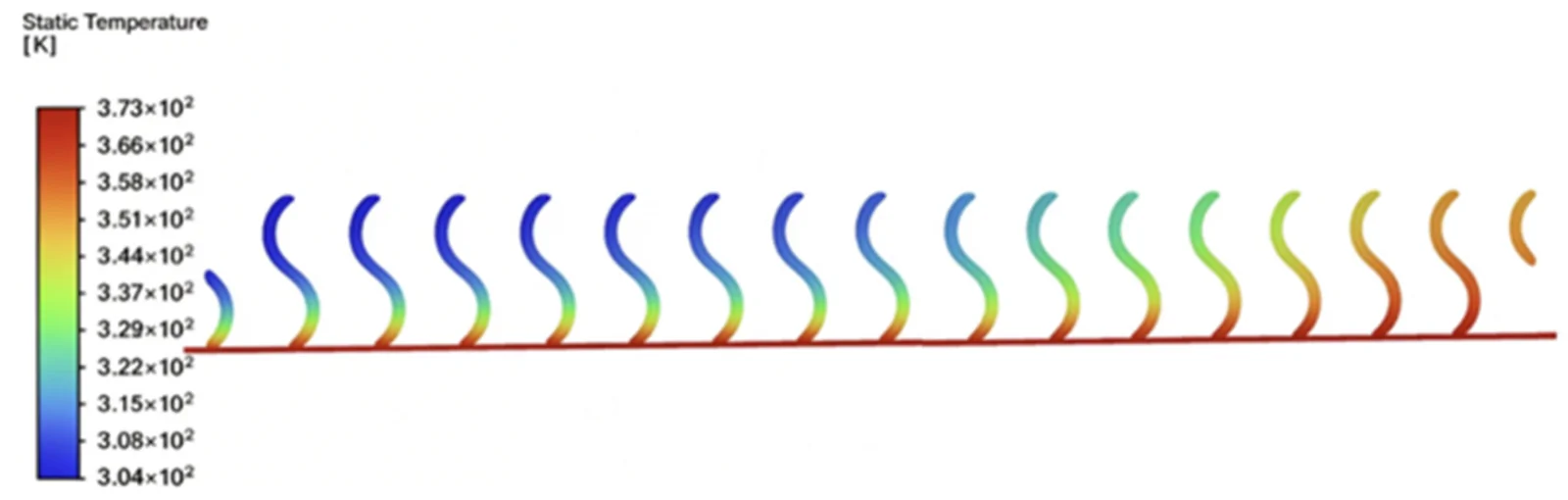
FVM model solid temperature curve on the y = 0 plane.

**Figure 40:**
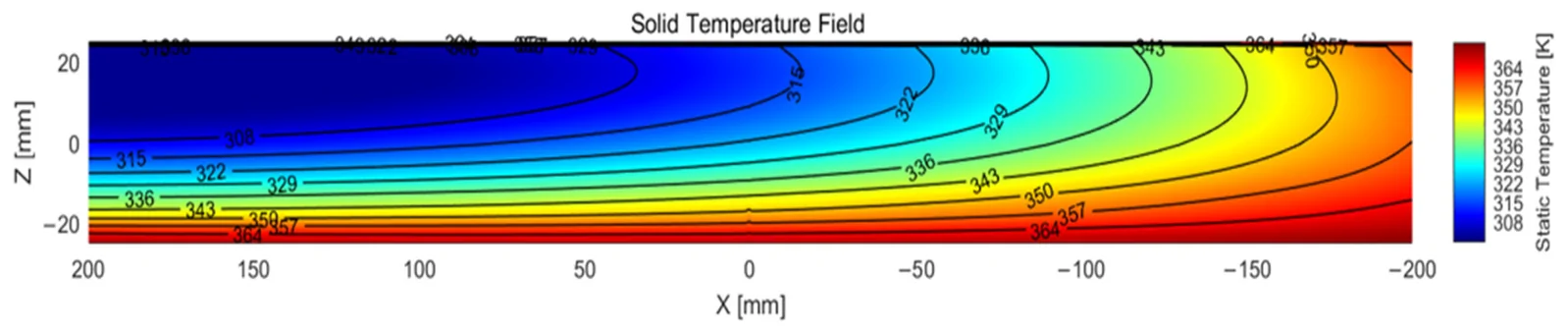
Equivalent model solid temperature curve on the y = 0 plane.

**Figure 41:**
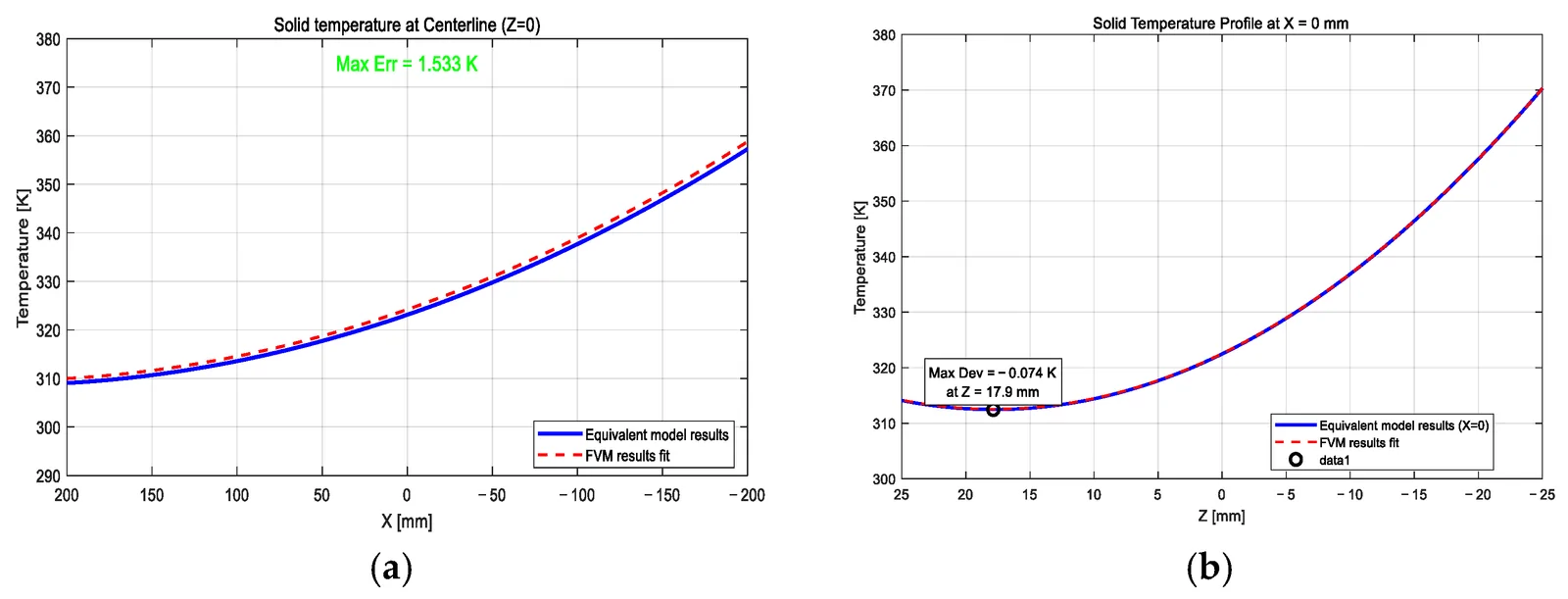
Solid temperature curve on the y = 0 plane. (**a**) Solid temperature along z = 0; (**b**) solid temperature along x = 0.

From the comparison between Figure 39 and Figure 40, it can be seen that the deviation between the equivalent model and the FVM model is very small. According to Figure 41a, the maximum deviation between the equivalent model and the FVM model in the solid temperature curve along the z = 0 is 1.533 K, with a relative error of 2.10%. From Figure 41b, it can be seen that the temperature curve along x = 0 has a smaller error of 0.074 K, with a relative error of 0.101%. These small errors prove the accuracy and effectiveness of the equivalent model.

For the 8 × 8 model, the proposed method employs approximately 8 million mesh cells, whereas the FVM approach generates 11,989,581 cells, reducing memory consumption by approximately one-third. The computational time of the proposed method was 2 min, compared to 42 min for the FVM, achieving a significant reduction in simulation time.

#### 3.3.4. Error Convergence Analysis

We conducted a statistical analysis of the Gyroid-type temperature errors of the fluid phase, solid phase and exported heat under two-dimensional flow with 4 × 4 cells, 6 × 6 cells and 8 × 8 cells filled, and plotted the error convergence in Table 6. From Table 6, it can be seen that under two-dimensional flow, the fluid-temperature and solid-temperature errors decrease as the array size increases from 4 × 4 to 8 × 8. The error of the fluid phase compared with that of the FVM model decreases from 0.79% to 0.63%, showing a downward trend, and the temperature error of the solid phase decreases from 1.76% to 0.33%, also showing a downward trend. We can also see that when the filling quantity increases from 4 × 4 to 8 × 8, the heat error exported in the planar flow is decreases from 2.71% to 1.01%.

#### 3.3.5. Temperature-Field Calculation Under Variable Porosity and Variable Velocity

**Figure 42:**
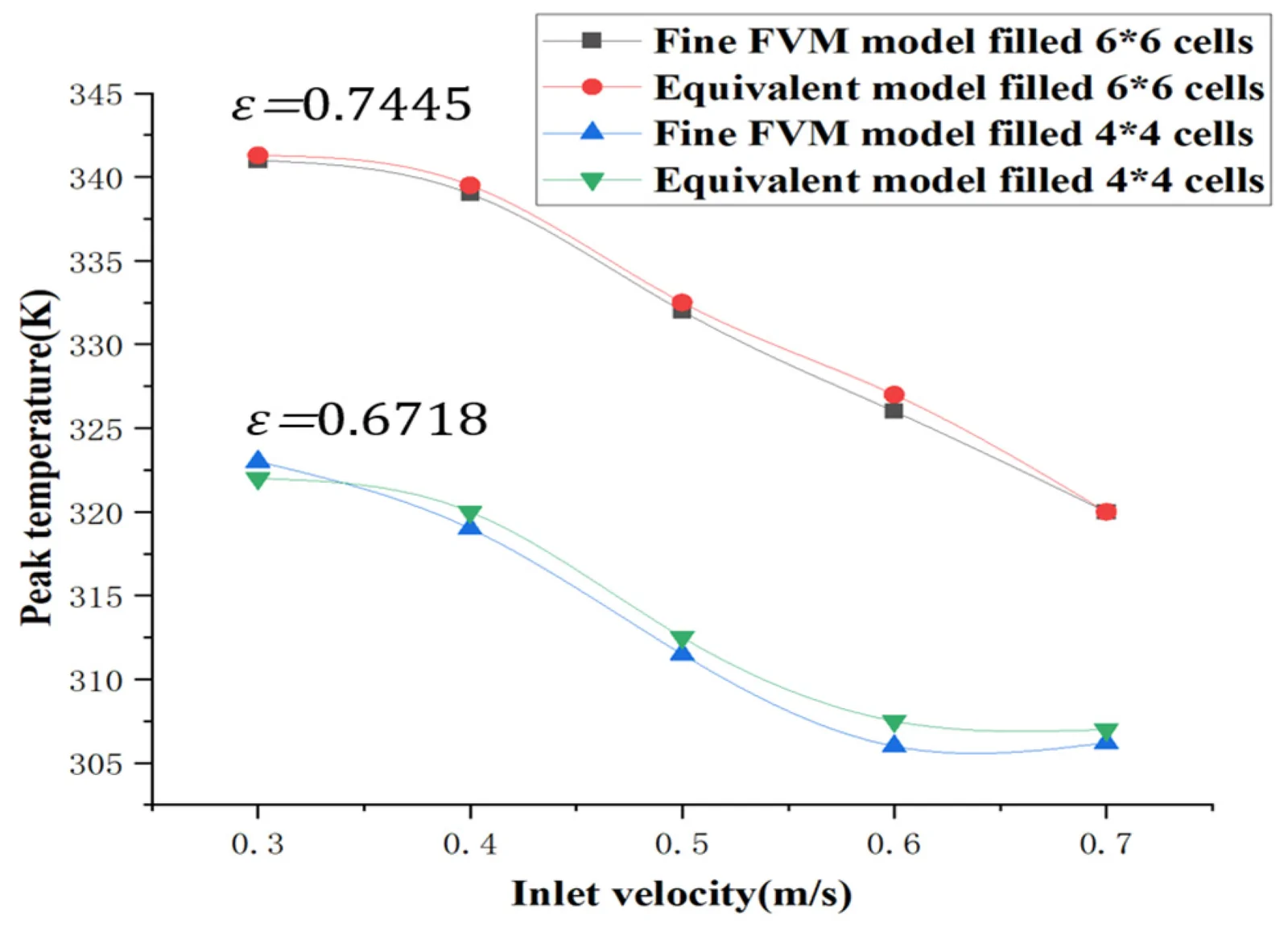
Typical peak temperature curves at maximum error for porosities of 0.6718 and 0.7445.

From Table 7, when 4 × 4 cells are filled, the maximum error of the dual-medium model is 2.05%, which occurs at a porosity of 67.18%, as shown in Figure 42. When the porosity is changed, the maximum error is further reduced for the case of 6 × 6 cell filling, resulting in an error of 1.37%, which occurs when the porosity is 74.45% or 88.41%. For the flow case filled with 8 × 8 cells, the maximum error has no change at a porosity range of 58.95–88.41%, which is 0.68%. From this perspective, the temperature error of cells decreases from 2.05% to 0.68%.

## 4. Conclusions

In this study, the Gyroid surfaces were modeled using nTopology software. For cases with a small number of filled unit cells, the model was imported into Fluent Meshing for mesh generation and subsequent FVM solution, yielding reasonable temperature-field distributions. Meanwhile, to address the temperature-field calculation for large-scale filling scenarios, the gyroid surface structures were equivalent to porous dual-medium models (comprising solid and fluid phases). Relevant formulas for solving the equivalent thermal conductivity tensor of the solid and fluid phases, as well as the equivalent volumetric heat transfer coefficient for solid–fluid heat transfer, were referenced to develop and solve a MATLAB-based temperature-field prediction program. The key achievements of this work are summarized as follows:

(1) A simplification strategy was proposed to model the Gyroid surface as a dual-medium model, enabling accurate and rapid calculation of the equivalent thermal conductivity tensors of the solid and fluid phases, as well as the equivalent volumetric heat transfer coefficient for solid–fluid heat transfer.
(2) A rapid temperature-field prediction program for equivalent models was developed using the three sets of coefficients obtained in (1). The verification of 4 × 4, 6 × 6 and 8 × 8 filled cells shows that the peak predicted temperatures of the solid and fluid phases are basically consistent with the FVM results, confirming the high accuracy and effectiveness of the prediction program.
(3) For the 4 × 4, 6 × 6, and 8 × 8 arrays examined, the fluid and solid-temperature errors decreased with increasing array size, while the heat-output error decreased from the 4 × 4 to the 8 × 8 scale. The results suggest improved agreement for larger arrays within the investigated range.
(4) The accuracy of the model for varying porosity and flow rate was also validated in this article.
(5) A rapid analysis method for the cooling heat transfer of Gyroid-type TPMS was established, achieving an excellent balance between analysis accuracy and computational efficiency.
(6) A fast analysis method based on the dual-medium model can be used to characterize the heat transfer performance of Gyroid heat exchangers under the studied porosity and flow rate range, that is, to quickly calculate the temperature-field of the heat exchangers during operation, instead of the time-consuming FVM calculation.
(7) The rapid analysis method proposed in this article has an overwhelming advantage in computational efficiency. The computation time and number of grids have both decreased, making it possible to conduct rapid parametric research and optimization design of Gyroid-type heat transfer structures on ordinary workstations.

This method provides a powerful technical approach for performance prediction and configuration optimization of Gyroid TPMS active heat exchange structures, and exhibits significant engineering application value.

## Figures and Tables

**Table 1:** Geometry model parameters.

Geometry Model	Unit Cell Size (${mm}^{3}$)	C	t (mm)	Porosity (%)
Gyroid structure	50 × 50 × 50	0	5	74.45

**Table 2:** Material constant.

Material	Density (kg/m^3^)	Thermal Conductivity (W/m·K)	Specific Heat Capacity (J/kg·K)
Aluminum alloy	2700	180	1800

**Table 3:** Boundary conditions.

Fluid	Inlet Velocity (m/s)	Inlet Temperature (K)	Heat Source (K)	Wall Condition
Water	0.4	300	373.15	Adiabatic

**Table 4:** Calculation of the solid phase equivalent thermal conductivity by the MATLAB program.

Solid Dimensions ($m^{3}$)	Solid Thermal Conductivity (W/(m·K))	Fluid Thermal Conductivity (W/(m·K))	Equivalent Thermal Conductivity (W/(m·K))
0.05×0.05×0.05	180	0.6	42.4

**Table 5:** Calculation of the fluid phase equivalent thermal conductivity by the MATLAB program.

Solid Thermal Conductivity (W/m·K)	Fluid Thermal Conductivity (W/m·K)	Equivalent Thermal Conductivity (W/m·K)
180	0.6	0.446426

**Table 6:** Error comparison for three Gyroid models of different sizes.

Model Size	4 × 4	6 × 6	8 × 8
Error in fluid temperature	0.79%	0.67%	0.63%
Error in solid temperature	1.76%	1.43%	0.33%
Error in exporting heat	2.71%	2.71%	1.01%

**Table 7:** The maximum fluid-phase error between the FVM model and the proposed model on the horizontal mid-plane when the porosity and flow rate change (K).

Model	Method		v	0.3 m/s	0.4 m/s	0.5 m/s	0.6 m/s	0.7 m/s	Max Error
		ε							
filled 4 × 4 cells	Fine FVM	58.95%		320.0	317.5	310.0	307.0	306.0	1.37%
	Equivalent method			320.0	318.5	310.5	307.5	306.2	
	Fine FVM	67.18%		323.0	319.0	311.5	306.0	306.2	2.05%
	Equivalent method			322.0	320.0	312.5	307.5	307.0	
	Fine FVM	74.45%		323.0	319.0	317.0	311.0	308.5	1.37%
	Equivalent method			323.0	320.0	317.5	311.0	308.0	
	Fine FVM	88.41%		323.0	320.0	319.0	310.0	305.0	0.68%
	Equivalent method			323.0	319.7	319.5	310.5	305.3	
filled 6 × 6 cells	Fine FVM	58.95%		337.0	330.0	323.5	320.0	314.0	0.68%
	Equivalent method			337.0	330.5	324.0	320.5	314.5	
	Fine FVM	67.18%		340.0	332.0	324.5	320.5	315.5	0.68%
	Equivalent method			340.0	332.5	325.0	321.0	315.5	
	Fine FVM	74.45%		341.0	339.0	332.0	326.0	320.0	1.37%
	Equivalent method			341.3	339.5	332.5	327.0	320.0	
	Fine FVM	88.41%		343.0	342.5	334.0	330.5	322.0	1.37%
	Equivalent method			343.0	342.5	334.0	329.5	321.0	
filled 8 × 8 cells	Fine FVM	58.95%		370.0	365.0	353.0	350.0	343.0	0.68%
	Equivalent method			370.5	365.5	353.0	350.5	343.0	
	Fine FVM	67.18%		371.0	367.0	353.0	350.0	345.5	0.68%
	Equivalent method			371.5	367.5	353.0	350.5	345.5	
	Fine FVM	74.45%		372.0	369.0	359.0	351.0	348.0	0.68%
	Equivalent method			372.0	368.5	359.5	350.5	347.5	
	Fine FVM	88.41%		373.0	369.5	360.0	351.0	348.0	0.68%
	Equivalent method			373.0	369.0	360.0	351.5	348.3	

## Data Availability

The raw data supporting the conclusions of this article will be made available by the authors on request.

## References

[1] Murillo J.D., Yandri V., Shah M., Liaw B., Bhatelia T., Sun B. (2026). Numerical investigation on hydrodynamic characterization of TPMS-based structures. Chem. Eng. Res. Des..

[2] Zhou H. (2023). A Study on the Convective Heat Transfer Characteristics of Triply Periodic Minimal Surface Structures with Gyroid, Diamond, and Iwp. Master’s Thesis.

[3] Deng S. (2024). Design and Study on Thermal Performance of TPMS Gradient Porous Structure. Master’s Thesis.

[4] Samad A., Lai W.-H. (2025). Experimental and simulation analysis of heat transfer in Gyroid heat exchangers with variable flow channels. Int. J. Heat Mass Transf..

[5] Zhang X., Li H., Dong H., Zhang Y., Sun M., Song Y. (2024). Thermal performance of phase change materials embedded in functional gradient triply periodic minimal surface structures. J. Energy Storage.

[6] Stallard S., Bergman T.L., Li X. (2025). The transient 1D dimensionless conduction heat rate approach applied to 3D TPMS structures. Int. J. Heat Mass Transf..

[7] Zhu P., Chen B., Sui L., Li H., Li K., Jian Y. (2025). Three-dimensional numerical simulation of melting characteristics of phase change materials embedded with various TPMS skeletons. Front. Energy.

[8] Wang J., Pu W., Zhao H., Qiao L., Song N., Yue C. (2024). Investigations on the heat transfer performance of phase change material (PCM)-based heat sink with triply periodic minimal surfaces (TPMS). Int. J. Heat Mass Transf..

[9] Wang J., Pu W., Zhao H., Qiao L., Song N., Yue C. (2024). Experimental and numerical investigations on the intermittent heat transfer performance of phase change material (PCM)-based heat sink with triply periodic minimal surfaces (TPMS). Appl. Therm. Eng..

[10] Wang J., Pu W., Zhao H., Qiao L., Song N., Yue C. (2025). Thermal characteristics study of a heat sink with embedded phase change material (PCM) in the triply periodic minimal surfaces (TPMS). Int. J. Therm. Sci..

[11] Wang J., Chen K., Zeng M., Ma T., Wang Q., Cheng Z. (2023). Investigation on flow and heat transfer in various channels based on triply periodic minimal surfaces (TPMS). Energy Convers. Manag..

[12] Wang J., Chen K., Zeng M., Ma T., Wang Q., Cheng Z. (2023). Assessment of flow and heat transfer of triply periodic minimal surface based heat exchangers. Energy.

[13] Tang W., Zou C., Zhou H., Zhang L., Zeng Y., Sun L., Zhao Y., Yan M., Fu J., Hu J. (2023). A novel convective heat transfer enhancement method based on precise control of Gyroid-type TPMS lattice structure. Appl. Therm. Eng..

[14] Tang W., Zhou H., Zeng Y., Yan M., Jiang C., Yang P., Li Q., Li Z., Fu J., Huang Y. (2023). Analysis on the convective heat transfer process and performance evaluation of Triply Periodic Minimal Surface (TPMS) based on Diamond, Gyroid and Iwp. Int. J. Heat Mass Transf..

[15] Gado M.G. (2025). Thermal management and heat transfer enhancement of electronic devices using integrative phase change material (PCM) and triply periodic minimal surface (TPMS) heat sinks. Appl. Therm. Eng..

[16] Gado M.G., Ookawara S., Hassan H. (2023). Utilization of triply periodic minimal surfaces for performance enhancement of adsorption cooling systems: Computational fluid dynamics analysis. Energy Convers. Manag..

[17] Gado M.G. (2024). Improving the charging performance of latent heat thermal energy storage systems using triply periodic minimal surface (TPMS) structures. J. Energy Storage.

[18] Qureshi Z.A., Elnajjar E., Al-Ketan O., Abu Al-Rub R., Al-Omari S.B. (2021). Heat transfer performance of a finned metal foam-phase change material (FMF-PCM) system incorporating triply periodic minimal surfaces (TPMS). Int. J. Heat Mass Transf..

[19] Qureshi Z.A., Al-Omari S.A.B., Elnajjar E., Al-Ketan O., Abu Al-Rub R. (2022). On the effect of porosity and functional grading of 3D printable triply periodic minimal surface (TPMS) based architected lattices embedded with a phase change material. Int. J. Heat Mass Transf..

[20] Qureshi Z.A., Al-Omari S.A.B., Elnajjar E., Al-Ketan O., Abu Al-Rub R. (2021). Using triply periodic minimal surfaces (TPMS)-based metal foams structures as skeleton for metal-foam-PCM composites for thermal energy storage and energy management applications. Int. Commun. Heat Mass Transf..

[21] Mulhi A., Dehgahi S., Waghmare P., Qureshi A. (2023). Dimensional assessment of uniformly periodic porosity primitive TPMS lattices using additive manufacturing laser powder bed fusion technique. Int. J. Adv. Manuf. Technol..

[22] Dutkowski K., Kruzel M., Walczak M. (2025). Heat Exchange and Flow Resistance in a Heat Exchanger Based on a Minimal Surface of the Gyroid Type—Results of Experimental Studies. Energies.

[23] Kruzel M., Dutkowski K., Bohdal T. (2025). Experimental Studies of Fluid Flow Resistance in a Heat Exchanger Based on the Triply Periodic Minimal Surface. Energies.

[24] Dutkowski K., Kruzel M., Rokosz K. (2022). Review of the State-of-the-Art Uses of Minimal Surfaces in Heat Transfer. Energies.

[25] Kruzel M., Bohdal T., Dutkowski K., Wołosz K.J., Robakowski G. (2025). The Heat Transfer Coefficient During Pool Boiling of Refrigerants in a Compact Heat Exchanger. Energies.

[26] Iwaniszyn M., Sindera K., Maszybrocka J., Jodłowski P. (2024). 3D-printed triply periodic minimal surface (TPMS) structures as catalyst carriers. Chem. Eng. Res. Des..

[27] Sindera K., Korpyś M., Iwaniszyn M., Gancarczyk A., Suwak M., Kołodziej A. (2022). New streamlined catalytic carriers of enhanced transport properties: Experiments vs. CFD. Chem. Eng. J..

[28] Korpyś M., Iwaniszyn M., Sindera K., Suwak M., Gancarczyk A., Kołodziej A. (2023). Flow phenomena in laminar flow through streamlined and sharp-edged short monolithic structures. Sci. Rep..

[29] Park J., Weibel J.A., Ziviani D. (2026). A topology optimization framework for fluid-to-fluid heat exchangers using triply periodic minimal surfaces (TPMS) unit cells as tunable building blocks. Int. J. Heat Mass Transf..

[30] Ohtani K., Kawabe H., Yaji K., Fujita K., Aute V. (2026). Homogenization-based optimization of wall thickness distribution for TPMS two-fluid heat exchangers. Int. J. Heat Mass Transf..

[31] Liu J. (2019). Research on the Flow Law of High Permeability Porous Media Based on Volume Average Method. Ph.D. Thesis.

[32] Ge H. (2017). Simulation Study on Macroscopic Segregation of Large Ingots Based on Volume Averaging Method. Ph.D. Thesis.

[33] Wang X., Jia J., Liu Z. Derive the generalized Darcy’s law for power-law fluids using the volume averaging method. Proceedings of the Fluid Mechanics Professional Committee of the Chinese Society of Mechanics Abstract Collection of Papers from the 8th National Conference on Fluid Mechanics Department of Thermal Science and Energy Engineering.

[34] Bo X., Hou J., Hou Y., Guo Z. (2025). Machine learning algorithm for two-phase flow model in porous media. Pure Math. Appl. Math..

[35] Li W., Wang Z., Tian H., Mou Y., Zhou Z., Jin Z., Qian J. (2025). Research progress on fluid sealing mechanism based on porous media model. Fluid Mach..

[36] Ji M., Gong S., Hou H. (2025). Construction of Natural Gas Hydrate Porous Media Model Based on Four Parameter Growth Method. Inn. Mong. Petrochem..

[37] Ye C., Xu S., Sun H., Lu J., Ye C., Liu W. (2025). Research on the rapid determination method of clay permeability coefficient by filtration under generalized Darcy’s Law. J. Railw. Sci. Eng..

[38] Qi C. (2019). Orthogonal nonlinear flow control equation. Pet. Drill. Prod. Technol..

[39] Cheng G., Liu S. (1996). Prediction of thermal conductivity of unidirectional fiber composite materials. J. Compos. Mater..

[40] Yao Q., Xia X. (2025). Research progress on homogenization methods for low dimensional functional composite materials. J. Appl. Mech..

[41] Zhu Q., Pishahang M., Caccia M., Kelsall C.C., LaPotin A., Sandhage K.H., Henry A. (2022). Validation of the Porous Medium Approximation for Hydrodynamics Analysis in Compact Heat Exchangers. Fluids Eng..

[42] Salazar R., Adrian E. (2022). Design of Functionally Graded Parts for Additive Manufacturing: Methods and Tools for Variable Density Triply Periodic Minimal Surfaces. Ph.D. Thesis.

